# Viral factors in influenza pandemic risk assessment

**DOI:** 10.7554/eLife.18491

**Published:** 2016-11-11

**Authors:** Marc Lipsitch, Wendy Barclay, Rahul Raman, Charles J Russell, Jessica A Belser, Sarah Cobey, Peter M Kasson, James O Lloyd-Smith, Sebastian Maurer-Stroh, Steven Riley, Catherine AA Beauchemin, Trevor Bedford, Thomas C Friedrich, Andreas Handel, Sander Herfst, Pablo R Murcia, Benjamin Roche, Claus O Wilke, Colin A Russell

**Affiliations:** 1 Center for Communicable Disease Dynamics, Harvard T. H Chan School of Public Health, Boston, United States; 2 Department of Epidemiology, Harvard T. H. Chan School of Public Health, Boston, United States; 3 Department of Immunology and Infectious Diseases, Harvard T. H. Chan School of Public Health, Boston, United States; 4 Division of Infectious Disease, Faculty of Medicine, Imperial College, London, United Kingdom; 5 Department of Biological Engineering, Koch Institute for Integrative Cancer Research, Massachusetts Institute of Technology, Cambridge, United States; 6 Department of Infectious Diseases, St. Jude Children’s Research Hospital, Memphis, United States; 7 Centers for Disease Control and Prevention, Atlanta, United States; 8 Department of Ecology and Evolutionary Biology, University of Chicago, Chicago, United States; 9 Department of Biomedical Engineering, University of Virginia, Charlottesville, United States; 10 Department of Molecular Physiology and Biological Physics, University of Virginia, Charlottesville, United States; 11 Department of Ecology and Evolutionary Biology, University of California, Los Angeles, Los Angeles, United States; 12 Fogarty International Center, National Institutes of Health, Bethesda, United States; 13 Bioinformatics Institute, Agency for Science Technology and Research, Singapore, Singapore; 14 National Public Health Laboratory, Communicable Diseases Division, Ministry of Health, Singapore, Singapore; 15 School of Biological Sciences, Nanyang Technological University, Singapore, Singapore; 16 MRC Centre for Outbreak Analysis and Modelling, School of Public Health, Imperial College London, London, United Kingdom; 17 Department of Infectious Disease Epidemiology, School of Public Health, Imperial College London, London, United Kingdom; 18 Department of Physics, Ryerson University, Toronto, Canada; 19 Vaccine and Infectious Disease Division, Fred Hutchinson Cancer Research Center, Seattle, United States; 20 Department of Pathobiological Sciences, University of Wisconsin School of Veterinary Medicine, Madison, United States; 21 Department of Epidemiology and Biostatistics, College of Public Health, University of Georgia, Athens, United States; 22 Department of Viroscience, Erasmus Medical Center, Rotterdam, Netherlands; 23 MRC-University of Glasgow Centre For Virus Research, Glasgow, United Kingdom; 24 IRD/UPMC UMMISCO, Montpellier, France; 25 Center for Computational Biology and Bioinformatics, Institute for Cellular and Molecular Biology, The University of Texas at Austin, Austin, United States; 26 Department of Integrative Biology, The University of Texas at Austin, Austin, United States; 27 Department of Veterinary Medicine, University of Cambridge, Cambridge, United Kingdom; University of Hong Kong, Hong Kong

**Keywords:** pandemic, risk prediction, influenza A, Human, Virus

## Abstract

The threat of an influenza A virus pandemic stems from continual virus spillovers from reservoir species, a tiny fraction of which spark sustained transmission in humans. To date, no pandemic emergence of a new influenza strain has been preceded by detection of a closely related precursor in an animal or human. Nonetheless, influenza surveillance efforts are expanding, prompting a need for tools to assess the pandemic risk posed by a detected virus. The goal would be to use genetic sequence and/or biological assays of viral traits to identify those non-human influenza viruses with the greatest risk of evolving into pandemic threats, and/or to understand drivers of such evolution, to prioritize pandemic prevention or response measures. We describe such efforts, identify progress and ongoing challenges, and discuss three specific traits of influenza viruses (hemagglutinin receptor binding specificity, hemagglutinin pH of activation, and polymerase complex efficiency) that contribute to pandemic risk.

**DOI:** http://dx.doi.org/10.7554/eLife.18491.001

## I**ntroduction**

Aquatic birds are the main reservoir of influenza A viruses in nature (Krauss and Webster, 2010). Influenza viruses from aquatic birds sporadically enter terrestrial bird and mammalian host populations and achieve sustained circulation in these new hosts (Vandegrift et al., 2010), sometimes after reassortment with influenza viruses already circulating in the new host (Webster et al., 1992). Adaptation of viruses from aquatic birds to mammals involves a change in tissue tropism from intestinal to respiratory epithelia (Hinshaw et al., 1979; Hénaux and Samuel, 2015).

Multiple influenza A subtypes—defined by the patterns of antibody recognition of two surface proteins, hemagglutinin (HA) and neuraminidase (NA)—circulate in avian species and swine at any given time. Among these, a number are known to cause sporadic zoonotic infections in humans (Peiris, 2009). More than one thousand human infections with avian influenza viruses were detected in the last decade, for example H5N1 and H7N9 (Qin et al., 2015) as well as swine influenza viruses, e.g. an H3N2 variant that spilled over into humans attending agricultural shows in the early 2010s, H3N2v (Jhung et al., 2013). In addition, zoonotic infections with other viruses from poultry or wild birds have occurred, including for example H7N7 (Fouchier et al., 2004), H10N8 (Wohlbold et al., 2015), H6N1 (Wei et al., 2013), H9N2 (Butt et al., 2005), and H5N6 (Yang et al., 2015); for more examples and a fuller discussion see (Short et al., 2015). The severity of zoonotic influenza A infections ranges from clinically inapparent (Gomaa et al., 2015; To et al., 2016) to fatal (de Jong et al., 2006; Gao et al., 2013).

Although secondary transmission can occur following some of these spillover events (Kucharski et al., 2014), only a very small proportion of them—four in the last hundred years, which seems to be close to the historical average (Patterson, 1986)—led to sustained person-to-person transmission with global spread (Box 1). There are 18 known HA types and 11 known NA types (Tong et al., 2013), which could theoretically be found in any combination. So far, sustained spread in humans has been limited to the H1N1, H2N2, and H3N2 subtypes (Kuiken et al., 2006), though it is possible that other subtypes circulated prior to 1918, the year of the first pandemic from which viruses are available for study (Worobey et al., 2014). Multiple virus–host interactions are necessary for replication and onward transmission; the differences in the genetic requirements to accomplish each of these interactions in humans versus other animals provide a barrier to sustained transmission following spillover (Russell et al., 2014).

10.7554/eLife.18491.002

Box 1. **Steps in pandemic emergence.**

For an avian-adapted strain of influenza A to become a pandemic strain, several events are required:

1. The avian-adapted strain must become sufficiently widespread in wild or domestic birds, swine or other reservoir species to expose at least one human to infection.
2. One or more humans must acquire infection from the reservoir species.
3. The infection must replicate sufficiently in a zoonotic case to produce infectious virus in respiratory or other secretions.
4. The infection must be transmitted to additional humans, avoiding an "early" termination of the transmission chain due to chance. Such early termination is a significant risk given the relatively low infectiousness of influenza and the moderate degree of overdispersion in the number of secondary cases infected by each case, both of which contribute to the probability that a transmission chain will terminate by chance (Lloyd-Smith et al., 2005; Lipsitch et al., 2003). It must also avoid extinction due to deliberate control efforts put in place by public health authorities (Ferguson et al., 2006; Merler et al., 2013).
5. Finally, the infection must spread beyond the local area to infect members of distant populations, a process accelerated by modern global travel (Cooper et al., 2006). This step and the one before are enhanced if the level of population susceptibility is high, as occurs when the surface proteins of the new strain are dissimilar to those on any currently or recently circulating human influenza A strains.

We know from serologic studies and human infections that several different influenza A viruses have achieved steps 1 and 2 at any given time (Gomaa et al., 2015; To et al., 2016). Steps 3 and/or 4 appear to be the rare, rate-limiting steps; that is, the conditional probability of achieving step 3 and 4 given the previous steps is low, so that sustained human-to-human transmission of a novel strain occurs a few times per century while zoonotic infections must occur thousands or more times per year. No case is known in which an influenza A strain has reached stage 4 but failed to reach stage 5, although it may have happened undetected.

The appearance -- by mutation or reassortment -- and selection of genetic changes that encode human-adaptive viral traits may be seen as a process that can accelerate or increase the probability of one or more of these steps (though there is no guarantee that a given change that enhances one of these steps will enhance all of them. This is why the detection of phenotypes associated with human adaptation in avian or zoonotic isolates of novel influenza A viruses is thought to correlate with increased risk of pandemic emergence. As we describe throughout the paper, the process of human adaptation need not be complete to initiate a pandemic, so it may continue to occur at various stages throughout this progression.

**DOI:** http://dx.doi.org/10.7554/eLife.18491.002

Experiments in ferrets have been used to measure viral transmissibility via respiratory droplets (in this review we use this term to refer to any transmission through the air between ferrets that are not in direct or indirect physical contact). Droplet transmission in ferrets is a useful, albeit imperfect, correlate of the potential of influenza strains to transmit efficiently in human populations (Buhnerkempe et al., 2015). For this reason, some have argued that there is a general phenotype of 'transmissibility by respiratory droplets in mammals' such that experiments to select for such transmission in droplets in ferrets are important models of the process of adaptation to human transmission (Imai et al., 2012; Herfst et al., 2012). This view is not universally shared (Palese and Wang, 2012). Starting from an zoonotic highly pathogenic avian influenza isolate from a human case of infection (or a reassortant of the HA from a different zoonotic H5N1 highly pathogenic avian influenza isolate, with the other segments from the 2009 pandemic H1N1 strain), it was shown that certain specific traits that had been previously associated with mammalian host adaptation were required to achieve respiratory droplet transmission. These ferret-transmission phenotypes in turn were associated with certain genetic changes relative to the original avian viruses (Imai et al., 2012; Herfst et al., 2012; Linster et al., 2014). These specific changes occur both in HA and in polymerase-complex proteins.

The rationale for these experiments was that, because the ferret model recapitulates many features of human infection, changes identified in adaptation to ferret transmission would also be important for adaptation to sustained transmission in humans (Davis et al., 2014; Schultz-Cherry et al., 2014), though this can never be known with certainty (Palese and Wang, 2012). Notably, viruses isolated from humans who were infected by contact with birds show some of these changes (Russell et al., 2012; Bi et al., 2015), particularly the change at amino acid 627 of the PB2 gene (Jonges et al., 2014; Chen et al., 2014; Fonville et al., 2013), which often affects polymerase complex efficiency (see below). This indicates that even the first generation of human infection from nonhuman hosts can initiate a process of host adaptation. It also indicates that not all the human-adaptive changes must be in place in the avian reservoir to initiate this process. Some human infections, including some zoonotic cases (de Jong et al., 2006; Chen et al., 2014, 2006; Sha et al., 2016) and some cases early in a pandemic (Rogers and D'Souza, 1989; Connor et al., 1994; Stevens et al., 2006; Glaser et al., 2005; Pappas et al., 2010), involve viruses that are not yet fully human-adapted (see below and Tables 2–4). The interpretation of some of these isolates is complicated by uncertainty about whether they were passaged in hen’s eggs at some point in their history.

Certain types of countermeasures against an influenza pandemic are effective only against one lineage of viruses – for example, creating stockpiles or seed stocks of vaccines against a particular subtype, or culling poultry infected with that subtype. It is not currently feasible to invest in such countermeasures against all viruses circulating in avian or other reservoirs, or even against all those causing known zoonotic cases. Therefore, there would be value in an accurate system to assess the relative pandemic risks posed by each virus and prioritize them for the development of such strain-specific countermeasures, while directing fewer resources to strains of lower concern (Kaplan et al., 2016). This consideration has motivated calls for comprehensive analysis of all available data to assess the threat to public health posed by these strains. One response is the CDC’s Influenza Risk Assessment Tool (IRAT) (Trock et al., 2015), which incorporates elements including properties of the virus, field and epidemiological findings, and attributes of the human population to provide a framework to differentiate among novel influenza viruses thought to possess pandemic potential. Such risk assessments can help focus pandemic prevention and response efforts on the viruses thought to pose the highest risk of pandemic spread (Davis et al., 2014), in the most worrisome cases providing a rationale for costly measures such as poultry culling or vaccine seed stock development, or even stockpiling of large quantities of vaccine. A guiding question of this article is to examine the degree to which it is justified to rely on measurements and predictions of viral genetic and phenotypic traits in prioritizing responses to particular viral subtypes and within-subtype lineages.

There are several hurdles to evaluating the accuracy of such predictions (Russell et al., 2014). Factors limiting our ability to identify high-risk viruses and predict the risk they pose include:

- limited surveillance of nonhuman influenza viruses, such that high-risk viruses may not be detected and hence cannot be assessed (Butler, 2012). Limitations include both the number, geographic and species diversity of hosts sampled, and the difficulty in sampling all genetic variants present in a given infection (Poon et al., 2016; Varble et al., 2014);
- failure to fully characterize some viruses that are detected (Hoye et al., 2010);
- imperfect public health systems lacking capacity to detect zoonotic infections presenting in patients (Sanicas et al., 2014; Simonsen et al., 2013);
- epistasis and other complexities that prevent straightforward prediction of viral traits from genotype (Russell et al., 2014; Kryazhimskiy et al., 2011; Gong and Bloom, 2014; Bloom et al., 2010; Wu et al., 2016; Raman et al., 2014; Tharakaraman et al., 2013; Gong et al., 2013);
- technological limitations in molecular modeling and phenotypic assays that limit confidence in predicting and measuring viral traits (Russell, 2014);
- uncertainties about the taxonomic level at which risk predictions should be made (Box 2);
- practical, ethical and cost limitations of animal transmission experiments, as well as some exceptions to the correlation between human transmissibility and droplet transmissibility in nonhuman animal models (Buhnerkempe et al., 2015);
- lack of data on immediate animal precursors of viruses that caused previous pandemics;
- multiple scales at which viral strains compete and hence experience selection (i.e. replication within hosts, transmission between hosts). Evolutionary theory for such multi-scale selection is incomplete. Viral fitness components are rarely measured at both scales for the same strain and are imperfectly correlated across scales (Gog et al., 2015; Park et al., 2013; Beauchemin and Handel, 2011);
- the role of stochastic events in the ecology and evolution of influenza viruses during and after host-switching to humans (Gog et al., 2015; Lloyd-Smith et al., 2015), including the potential for transmission bottlenecks to either promote or inhibit emergence of human-adapted viruses (Varble et al., 2014; Moncla et al., 2016; Wilker et al., 2013; Zaraket et al., 2015).

10.7554/eLife.18491.003

Box 2. **Granularity of pandemic risk prediction – for what taxonomic level does it make sense?**

Determining the appropriate taxonomic level for influenza virus risk assessment is a challenging endeavor. Influenza virus subtype is a convenient classification but there can be substantial variation in estimable risk within subtype. For example, H5N1 viruses can be roughly segregated into high pathogenicity and low pathogenicity phenotypes with the high pathogenicity variants generating substantially greater concern for both human and animal populations. Even within the high pathogenicity H5N1 variants, risk to animal populations and potential for adaptation to humans is likely to vary by phylogenetic lineages or clades of viruses. Much of the difficulty for predicting the threat posed by subtypes or coarse grained concepts of virus variants stems from two factors: first, a lack of understanding of how genetic context affects the ability of a virus to adapt for efficient spread in humans; and second, the critical, and geographically variable, role of ecology in determining likelihood of cross species transmission.

Phylogenetic clade is a practical unit for risk prediction. However, in species where reassortment is frequent, phylogenetic clade must be considered on a gene by gene basis. The definition of phylogenetic clades can be challenging and arbitrary, but recent efforts to develop a unified nomenclature for highly pathogenic H5N1 viruses may offer a transferrable framework for the classification of other viruses (Smith and Donis, 2015; WHO/OIE/FAO H5N1 Evolution Working Group, 2008). Clades of viruses circulating in poultry, swine or other domestic animals with extensive human interactions should be prioritized for surveillance and further study. Foundational efforts are required to assess the diversity of viruses present in these animal populations, particularly for low pathogenic avian influenza viruses. Further study will then be required to assess the abundance and prevalence of different virus subtypes and clades, along with geographic spread and overlap with ecological risk factors(Hill et al., 2015; Gilbert et al., 2014), e.g. live animal markets, cohabitation of humans and animals, and biosecurity in animal processing facilities.

Antigenic characterization of animal influenza viruses should form part of a comprehensive risk assessment, particularly of viruses from swine and possibly dogs. Swine influenza virus diversity is driven in large part by introductions of viruses from humans to swine (Nelson et al., 2015, 2014; Lewis et al., 2016). The substantial antigenic diversity of viruses circulating in swine and antigenic differences with viruses circulating in humans poses an ever increasing risk for re-introduction into humans. Much of the antigenic variation in swine has a strong relationship to phylogenetic clade (Lewis et al., 2016). Similarly, the high contact rates between humans and dogs, combined with increased circulation of H3N2 canine influenza viruses, may present increasing opportunities for reassortment (Na et al., 2015) and for zoonotic infections (Flanagan et al., 2012).

**DOI:** http://dx.doi.org/10.7554/eLife.18491.003

These difficulties are exacerbated by the fact that influenza pandemics are rare events, and that risk assessments are not yet made with enough quantitative precision to formally evaluate their practical application. Even perfect information about the viral determinants of pandemic risk might only be enough to distinguish between strains with a low risk of causing a pandemic (say, 0.1% per year) and those with an extremely low risk (say, less than 0.01% per year), with unpredictable ecological or evolutionary contingencies determining which of these low-probability events will actually occur. One such contingency is that an avian influenza virus could acquire one or more of the determinants of pandemic potential by reassorting with a human seasonal influenza virus.

With only one pandemic every few decades, the data set for testing the prediction of such rare events is inadequate, a problem that challenges predictions in many fields beyond infectious diseases (King and Zeng, 2001; Hansson, 2006). Evolutionary events in which a strain increases human-to-human transmissibility, but not enough to spark a pandemic, are extremely hard to observe, but if we could do so it would increase our ability to characterize the process of adaptation (Kucharski et al., 2015).

**Figure 1.**
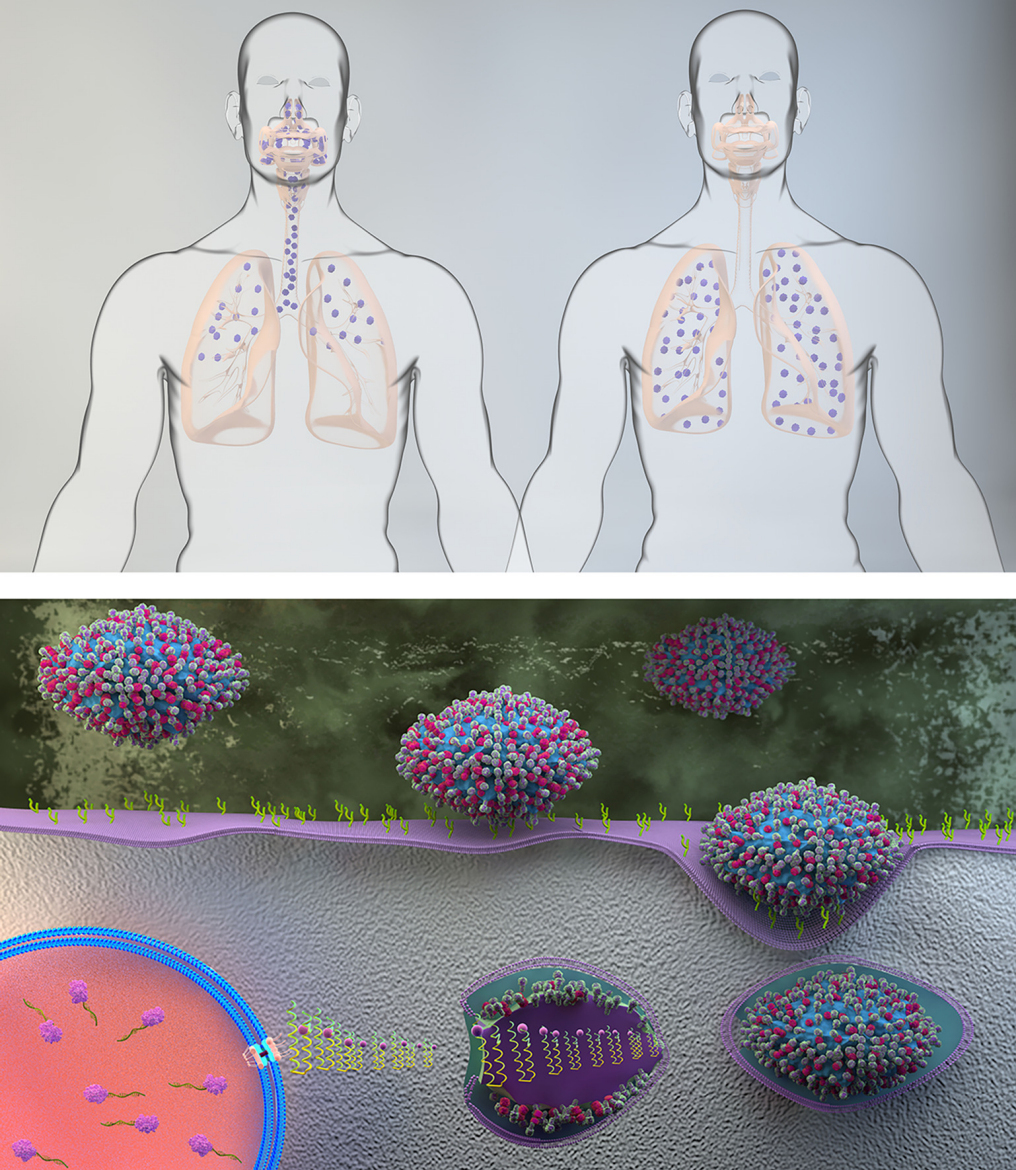
Key phenotypic traits for the adaptation of avian influenza viruses to replicate efficiently in humans. (**A**) A switch in receptor binding preference from avian-like (α2,3-linked sialic acid) to human-like (α2,6-linked sialic acid) receptors. The human form on the left shows the typical distribution of human adapted influenza viruses determined by their receptor binding preference for a2,6, linked SA that is predominantly expressed in the upper respiratory tract but also in the lungs. The human form on the right shows that infection with avian influenza viruses is concentrated in the lungs where their preferred a2,3 linked SA receptor is expressed. (**B**) Lower HA pH of activation and increased polymerase complex efficiency. Free-floating viruses that enter the human respiratory tract (upper part of figure) encounter mucus and a mildly acidic extracellular environment that act as innate barriers to virus infection. If NA is able to desialylate decoy receptors on mucus and HA has a sufficiently low pH of activation, then the virus particle may reach the apical surface of the respiratory epithelium intact. There through a multiplicity of interactions between HA and cell-surface sialic acid, the virus enters the target cell. After the virus is internalized, it passes through the endosomal pathway where the pH is progressively decreased. The low pH of the endosomal environment triggers an irreversible conformational change in HA that fuses the viral and endosomal membranes and ultimately results in the release of virus genetic material in the form of the viral ribonucleoprotein complex (vRNP) into the cell cytoplasm. The eight vRNPs are subsequently imported into the cell nucleus by interactions between the vRNPs and cellular nuclear import machinery. Inside the nucleus the virus polymerase complex replicates the virus genome in conjunction with co-opted cell proteins. **DOI:** http://dx.doi.org/10.7554/eLife.18491.005

Despite these challenges, there has been tremendous interest and investment in making and using such predictions, and a number of new ideas to improve predictions are in various stages of development (Box 3). Building on the findings of a previous workshop (Russell et al., 2014), we considered in detail the present state of knowledge concerning three phenotypic traits: HA receptor binding specificity, and HA pH of activation, and polymerase complex activity, (Figure 1). These were chosen from a longer list of candidate traits (Table 1) because they span the viral life cycle (Figure 1) and their role in host adaptation has been extensively studied. All three are believed to be required for an influenza virus to cause a pandemic; consistent with this assumption, all three traits have been present to some degree in the earliest viruses isolated in pandemics since the 20th century, though some have been enhanced by subsequent evolution during seasonal transmission in humans. Moreover, for each of these three traits, viruses isolated from avian hosts typically do not show the mammalian-adapted phenotype, reflecting divergent selection pressures in the two classes of hosts (Tables 2, 3, and 4). All three traits changed in the adaptation of zoonotic H5 influenza viruses to droplet transmission in ferrets (Imai et al., 2012; Linster et al., 2014). We emphasize that each of these traits is quantitative, and that human-adaptation is not a threshold criterion but a continuum; in this review when we speak of human adaptation we mean a tendency to be better adapted to humans, rather than an absolute yes-or-no property.

10.7554/eLife.18491.004

Box 3. **Novel approaches to identifying genomic predictors of traits and transmission phenotypes.**

The advent of inexpensive, large-scale sequencing, combined with improved computing power and novel algorithms to interpret nucleotide and protein sequences, have generated new approaches to characterizing the genotype-trait and genotype-transmission phenotype maps in influenza viruses. Some are well-established, while others are under active development. They include:

*Protein structural analysis to identify properties of individual amino acid residues and pairs of residues.* A number of approaches have been devised to make use of databases of genome sequences and inferred protein sequences of influenza virus isolates, alone or in combination with metadata on the source (species), date of isolation and passage history of the isolates.

Characterizing the predictors – at the level of individual amino acid residues within a protein – of variability or conservation can assist in identifying the major selection pressures on that protein. Evolutionary analysis of the predictors of high rates of nonsynonymous substitutions within HA showed solvent accessibility and proximity to the sialic acid receptor binding site are the strongest predictors of high nonsynonymous evolutionary rates (Meyer and Wilke, 2015). Comparisons of residue-specific evolutionary rates in avian and human lineages can help to assess which sites are specifically involved in human adaptation and which may be evolving in avian reservoirs with potential consequences for human adaptation (Meyer et al., 2013).

*Innovative use of metadata* associated with sequences deposited in databases will be required to ensure that computational inferences from these databases are reliable. For example, methods that aim to identify sites under positive selection in the HA protein frequently find regions or sites that seem to contradict experimental evidence (Meyer and Wilke, 2015; Tusche et al., 2012; Kratsch et al., 2016). Several of these apparent contradictions can be resolved by accounting for viral passaging. For example, passaging in regular MDCK cells produces a strong signal of positive adaptation underneath the sialic-acid binding site; this signal is entirely absent in unpassaged virus or virus passaged in SIAT1 MDCK cells (McWhite et al., 2016). At the same time, passage bias mutations are assumed to increase fitness of the strain in the respective species and are often necessary to grow in culture at all. Therefore, sites associated with isolates passaged in mammalian cultures vs. those passaged in embryonated hen’s eggs have the potential to further identify sites associated with mammalian or human adaptation.

Metadata can also help to point to individual amino acids associated with human adaptation. For example, one proposed computational approach is to find potentially zoonotic human-isolated sequences when the majority of their database hits from preceding years were of animal origin. This serves, on one hand, as systematic survey to derive lists of times and places of likely zoonotic events and, on the other hand, provides close sequence pairs of zoonotic human and their putative animal precursors. In those pairs, common sites that repeatedly changed from the animal to the human zoonotic isolates could be reasoned as being involved in human adaptation. Combining these sites with those from passage changes, provides strong evidence for the involvement of a particular site in host adaptation.

*Network analysis* of the level of sequence covariation of pairs of residues among protein sequences in the database has led to the identification of groups of mutually covarying sites, which have been used to define features of the HA protein that play a role in determining glycan receptor usage (Kasson et al., 2009; Kasson and Pande, 2009). Complementary to such covariation analysis is the analysis of predicted molecular interactions. Using X-ray co-crystal structures or modeled structural complexes of HA-glycan receptors, molecular features have been defined as distinct networks of inter-residue interactions involving key residues that make contacts with the different glycan receptor topologies. These features go beyond hallmark residue analyses and more accurately predict how amino acid variations in the receptor binding site impact the inter-residue interactions and glycan receptor binding specificity (Raman et al., 2014). Similarly, network analysis of amino acid residues predicted to have significant interactions has shown that antigenic sites on the HA interact with residues controlling glycan receptor binding specificity, and that changes in these antigenic residues can then lead to changes in receptor-binding affinity (Soundararajan et al., 2011).

It seems likely that as these different lines of evidence – structural location, biophysical interaction, sequence covariation, sequence evolutionary rates, association with zoonotic or in vitro adaptation, etc.—begin to be understood at the resolution of individual amino acids within an influenza protein, such overlapping approaches will yield clearer understanding of the genetic and structural bases of host adaptation to human infection and transmission. A significant step toward such integration is the recent release of the FluSurver online tool which automatizes influenza sequence and structure analysis and highlights mutations that could alter the discussed traits based on extensive literature-derived genotype to phenotype lists, structural visualization of the mutation positions and their geographic and temporal frequency of occurrence and co-occurrence for epidemiological relevance (http://flusurver.bii.a-star.edu.sg and directly from within GISAID http://www.gisaid.org). In particular, the tool has been successful in picking up mutations affecting host receptor binding (Maurer-Stroh et al., 2014) as well as pH dependency (Cotter et al., 2014; Maurer-Stroh et al., 2010). However, also in this approach, annotations of the effects of mutations are based on inference from similarity to mutations studied in specific sequence contexts, which in most cases will not be identical to the investigated input sequences.

*Association studies.* Understanding the genetic basis of adaptive phenotypic change is a central goal in biology, and influenza poses special challenges and advantages relative to other organisms. Association studies have begun mapping the genomes of *Arabidopsis thaliana* to over 107 quantitative traits and the genomes of humans to over 100,000 (Bergelson and Roux, 2010; Leslie et al., 2014). These studies often investigate genetic variation at the scales of single nucleotide polymorphisms, alleles, and loci. Motif-based approaches have already proven useful in influenza (e.g., the insertion of multiple basic amino acids indicates highly pathogenic H5 and H7), and such simple, robust correlations simplify the prediction of phenotypic traits. Recent investigations of influenza (Thyagarajan and Bloom, 2014; Ashenberg et al., 2013; Pinilla et al., 2012) have shown that many mutations have roughly consistent impacts across diverse backgrounds. A complication of all association studies is confounding from genetic linkage and diverse environmental selective pressures. Although influenza’s genes might be tightly linked over short time scales, the virus evolves quickly, and many traits can be assumed to be under stabilizing selection. Thus, association studies that appear statistically impractical now may be feasible with a few more years of expanded surveillance.

As reviewed here, however, influenza often breaks simple genetic rules, perhaps due to epistasis (e.g., [Bloom et al., 2010]). High-dimensional genotype-phenotype relationships obscure simple correlations from association studies. A relevant lesson comes from The Cancer Genome Atlas (TCGA), which amassed sequences from thousands of diverse tumors to investigate the mutations leading to different cancer types. Although metastatic cancers are typically conceptualized as possessing six main phenotypic traits (Hanahan and Weinberg, 2011), TCGA revealed that the genetic commonality among tumors of any given type is shockingly low (Kandoth et al., 2013; Ledford, 2015). Human genomes are much larger and more complex than influenza’s, however, and so it is possible that an influenza atlas might reveal more patterns, which could inspire hypothesis-driven experiments (Weinberg, 2010).

*High-throughput, large-scale screens of mutational effects on hemagglutinin receptor binding*. Binding of upper-respiratory-tract glycans by the influenza virus hemagglutinin is one of the best-understood ingredients in making a virus capable of efficient human transmission. Yet the viral sequence determinants of this trait have been mapped only for a limited number of variants. A systematic screening strategy to scan the genetic “landscape” for sequences with a preference for human glycan receptors might include four components: (1) selection of viral genetic background, (2) large-scale mutagenesis, (3) screening and selection, and (4) confirmatory assays. Because both mutations near and far from the sialic-acid-binding site on hemagglutinin have been shown to alter glycan specificity, this should be based on a minimally biased approach to mutagenesis: screening combinations of all possible substitutions at all hemagglutinin residues that are not absolutely conserved across known subtypes. Critical considerations include choice of viral genetic background (both subtype and strain identity), extent of combinatorial screening (if conserved sites are omitted, every mutant containing changes at up to 4 simultaneous sites could be screened with substantial effort), and design of highly parallel screening, selection, and confirmatory assays. The mutagenesis and screening involved would be extremely large in scope: (before eliminating conserved residues, all 4-site mutnats ~[550 residues x 20 amino acids]^4^ = 1.4 x 10^16^ variants for each subtype tested). However, some computational pre-screening to narrow the set of residues tested combined with contemporary mutagenesis and screening technologies such as deep scanning codon mutagenesis (Thyagarajan and Bloom, 2014; Bloom, 2015; Fowler and Fields, 2014) make such an endeavor feasible.

**DOI:** http://dx.doi.org/10.7554/eLife.18491.004

**Table 1.** Influenza virus adaptations that appear to be required for human-to-human transmission. **DOI:** http://dx.doi.org/10.7554/eLife.18491.006

Trait	Adaptation
HA receptor binding specificity	Preference for α2,6-linked mammalian sialic acid receptors over α2,3-linked avian ones (Russell et al., 2006)
HA pH of activation	HA avoids extracellular inactivation and undergoes conformational changes leading to membrane fusion at appropriate pH for human cells (5.0–5.4 or perhaps 5.5) (Russell, 2014)
Polymerase complex efficiency	Efficient replication in human cells (Cauldwell et al., 2014; Naffakh et al., 2008)
Virus morphology	Filamentous morphology associated with several adaptations to mammals (Seladi-Schulman et al., 2014; Seladi-Schulman et al., 2013; Campbell et al., 2014; Beale et al., 2014)
Length of NA stalk	Longer stalk of NA required to penetrate human mucus and deaggregate virions (Blumenkrantz et al., 2013)
Antagonism of interferon production	Species-specific binding of the NS1 protein to host factors (Rajsbaum et al., 2012)
HA-NA “balance”	Substrate selectivity and catalytic rate of NA are calibrated to “balance” avidity of HA for the cell-surface glycan receptor (Zanin et al., 2015; Baum and Paulson, 1991; Yen et al., 2011; Handel et al., 2014)

**Table 2.** Hemagglutinin receptor binding preference and examples of viruses isolated from avian and human hosts showing preference for human or avian receptors, or mixed preference. Yellow-shaded cells show concordance between expected and observed properties. **DOI:** http://dx.doi.org/10.7554/eLife.18491.007

	Avian receptor preference	Mixed receptor preference	Human receptor preference
Expected sequence, trait. Hallmark residues HA 190, 225 (H1,H3), 226 (H3); many others	Preferential binding to α2,3 sialylated glycans. HA 190Glu, 225Gly, 226Gln	Similar binding to both classes of glycans	Preferential binding to α2,6 sialylated glycans. HA 190Asp, 225Glu, 226Leu
Found in avian isolates	Many examples: many avian isolates of subtypes H5N1 (Russell et al., 2012; Yamada et al., 2006), H2 (Connor et al., 1994) and H3 (Connor et al., 1994)	avian isolates of H5N5 (Li et al., 2015), North American H7 (Belser et al., 2008), H7N9 (Schrauwen et al., 2016), as well as examples from H2 (Connor et al., 1994; Liu et al., 2009) and H3 (Connor et al., 1994)	Some H9N2 avian isolates (Matrosovich et al., 2001; Li et al., 2014)
Found in human isolates	H5N1 zoonotic isolate (Imai et al., 2012; Yamada et al., 2006); one H1N1 isolate from 1957 (Rogers and D'Souza, 1989)*; some early H2N2 pandemic/seasonal isolates (Connor et al., 1994; Pappas et al., 2010; Matrosovich et al., 2000)*	Early H1N1 pandemic isolates from 2009(Childs et al., 2009) and 1918 (Stevens et al., 2006; Glaser et al., 2005); several H1N1 from the 1918-1956 period (Rogers and D'Souza, 1989)*; early H2N2 isolate from 1958 (Pappas et al., 2010); human isolate of zoonotic H7N9 (Xiong et al., 2013b)	Many examples: H1N1 post-1977 (Rogers and D'Souza, 1989); early H1N1 pandemic isolates from 2009 (Chen et al., 2011) and 1918 (Stevens et al., 2006; Glaser et al., 2005); most human H2 and H3 seasonal isolates (Connor et al., 1994; Matrosovich et al., 2000)

*These anomalous results are speculated by the authors to be possibly, or even probably the result of laboratory adaptation to egg passage and may not reflect the properties of the primary isolate. A possible counter to this interpretation is that it is seen only in the earliest isolates from human pandemic viruses, while nearly all isolates from after the pandemic year, which should also have been passaged in eggs, show human-adapted phenotypes.

**Table 3.** Hemagglutinin pH of acivation.Yellow-shaded cells show concordance between expected and observed properties. **DOI:** http://dx.doi.org/10.7554/eLife.18491.008

	Avian-adapted for transmissibility	Human-adapted for transmissibility
Expected trait	pH of fusion >5.4 (Reed et al., 2010)	pH of fusion 5.0-5.4 (5.5 for early H1N1pdm) (Russier et al., 2016)
Found in avian isolates	Avian H1-H4, H11 isolates (Galloway et al., 2013; Russier et al., 2016; DuBois et al., 2011; Reed et al., 2010)	Avian H5, H8, H9,H10,H14,H15 isolates (Galloway et al., 2013)
Found in human isolates	H5N1(Imai et al., 2012; Linster et al., 2014) and H7N9 (Schrauwen et al., 2016) human zoonotic isolates with pH ≥5.6. One human H1N1 (2008) isolate.	Human isolates of H1N1 (1918 and 2009 lineages), H2N2, H3N2 (Galloway et al., 2013)

**Table 4.** Polymerase complex efficiency; entries list amino acid at PB2 627, though other residues are clearly relevant to this trait.Yellow-shaded cells show concordance between expected and observed properties. **DOI:** http://dx.doi.org/10.7554/eLife.18491.009

	Avian-adapted	Human-adapted
Expected trait	Low efficiency in mammals, PB2 590/591 G/Q, 627E, 701D	High efficiency in mammals, PB2 590/591 S/R, PB2 627K, 701N;
Found in avian isolates	Nearly all avian sequences in databases as of 2005 (Chen et al., 2006)	A few entries in databases show sequences associated with human adaptation as of 2005 (Chen et al., 2006)***
Found in human isolates	zoonotic H9N2 (Chen et al., 2006); some zoonotic H5N1 (de Jong et al., 2006; Chen et al., 2006); some zoonotic H7N9 (associated with milder course) (Sha et al., 2016); one zoonotic H5N6 (Zhang et al., 2016)**	Pandemic and seasonal H1N1, H2N2, H3N2 from 1918-2008 (Cauldwell et al., 2014); some zoonotic H5N1 (Chen et al., 2006); some zoonotic H7N9 (associated with more severe course) (Sha et al., 2016); H1N1pdm (Herfst et al., 2010)*; one zoonotic H5N6 and one zoonotic H10N8 (Zhang et al., 2016)

* the role of amino acids 590 and 591 in adaptation was not recognized until after the 2009 strain had already emerged (Mehle and Doudna, 2009); it has the residues associated with avian adaptation at sites 627 and 701 that were known at that time (Herfst et al., 2010).

** complete sequence information not given in the paper.

*** the rarity of these raises questions about possible sequencing errors.

This review starts with a summary of our knowledge about the role of each of the three functional traits in conferring pandemic potential on a virus strain. Following these case studies, we draw some generalizations about the prospects of predicting pandemic risk from virus genotype or from assays of particular viral traits. For each trait we present a table showing the degree to which the sequence changes or phenotypic properties associated with avian or human adaptation are present in isolates from birds and humans, respectively. If the avian traits were always found in avian isolates and human traits always in human isolates, only the shaded cells on the main diagonal would be filled. In such a case, however, it is hard to see how viruses would ever make the jump from birds to humans, since so many traits would have to change simultaneously, and indeed the off-diagonal cells are not empty. Finding avian-adapted traits in viruses isolated from humans most often occurs in zoonotic cases, showing that not all human-adapted traits are required for the first human infection. In some cases there are also viruses isolated from humans after a pandemic starts that retain some degree of avian-like traits, and we discuss these in more detail in the text -- these represent the greatest challenge to use of genotypic or phenotypic information for pandemic prediction because they run the risk of false negatives. The other off-diagonal cell, which represents avian isolates with some human-like traits, simply shows that some circulation of viruses in birds is possible without the classical 'avian' phenotypes. How this happens is a phenomenon worthy of further study. We conclude with some recommendations for future research and for the practice of pandemic risk assessment.

## Trait 1: Hemagglutinin receptor binding specificity

### A. Definition of the trait

Attachment of an influenza virus to a host cell requires binding of the viral HA to a sialylated glycan receptor (sialic acid) on the surface of the host cell. Cells of the avian gut and a minority of cells in deep lung in mammals predominantly express receptors terminated with an α2,3-linked sialic acid: hereafter, α2,3 glycans or *avian receptors* (Russell et al., 2006; Gambaryan et al., 1997; van Riel et al., 2006; Shinya et al., 2006). By contrast, in humans and other mammals, upper respiratory epithelial cells express mainly glycan receptors terminated by α2,6-linked sialic acid: α2,6 glycans or *human receptors* (Stevens et al., 2006; Shinya et al., 2006; Chandrasekaran et al., 2008). The human upper respiratory epithelium is the primary target site for infection of human-adapted viruses, and infection at this site is thought to be a prerequisite for efficient human-to-human transmission via respiratory droplets. Thus, it appears that human adaptation of an HA is associated with a switch in its binding preference from avian to human receptors. Receptor binding is not either-or; human-adapted influenza virus HA may show some binding to avian receptors, and vice versa.

Receptor binding preference is defined as the ratio of affinity (or avidity) of an HA molecule for an α2,6 glycan relative to that for an α2,3 glycan, with higher values associated with greater human adaptation. The evolution of receptor binding specificity is driven by the host environment, with selection for specificity during the infection process within a host and during the process of transmission. The error-prone replication of influenza genomes can facilitate rapid emergence of viruses with amino acid substitutions that alter the receptor binding characteristics of the HA (Lakdawala et al., 2015). Increased transmissibility may result from mammalian receptor adaptation, either because the virus shedding form the infected donor host is increased, or because the ability of virus to infect the recipient host at a low dose is enhanced, or for both of these reasons. Recent experimental evidence in ferrets implicates the soft palate as an important site of selection for α2,6 specificity (Lakdawala et al., 2015).

### B. Genetic and structural determinants of hemagglutinin-receptor interactions

Preference for binding human or avian glycan receptors is determined by the structure of the viral HA. Except for a few conserved amino acids in the sialic acid receptor binding pocket, the influenza HA has considerable structural plasticity to evolve variation at the rim of the pocket to engage different sialic acid linkages. Importantly, antigenic regions of the HA are located nearby regions that determine receptor-binding preference, meaning that selection for antigenic escape may be constrained by the need to maintain receptor preference (Koel et al., 2013). More speculatively, selection for changes in receptor preference might also alter recognition of the HA by host antibodies.

**Conformation of hemagglutinin as a determinant of receptor binding preference**. Although the co-crystal structures of HA and sialylated glycans have not been solved for all pairs, there is evidence that avian- or human-adapted HA bind to different conformations of the avian and human receptors: the *cis* conformation of human receptors and the *trans* conformation of avian receptors (Stevens et al., 2006; Ha et al., 2001, 2003; Gamblin et al., 2004; Liu et al., 2009; Xu et al., 2010; Yang et al., 2010; Lin et al., 2012; Yang et al., 2012; Zhang et al., 2013). This finding has led to the concept of “hallmark” residues within the receptor-binding site of avian- and human-adapted HAs. Avian-adapted HAs typically carry Glu at position 190, Gln at position 226, and Gly and position 228 (H3 numbering), and the Gln226->Leu, Gly228->Ser substitutions have been associated with a switch to human receptor preference in HAs of H2, H3 (Matrosovich et al., 2000), and H5 (Chutinimitkul et al., 2010) viruses. In H1 HA, Glu190→Asp and Gly225→Asp have been considered as hallmark amino acid changes to switch receptor specificity leading to greater human adaptation (Glaser et al., 2005; Tumpey et al., 2007). The determinants of specificity are reviewed in much more detail in (Paulson and de Vries, 2013).

**Additional structural features involved in receptor binding preference**. The *cis* and *trans* definition of glycan conformation does not fully describe HA binding to a range of structurally diverse glycans displayed on human respiratory cells and tissues (Chandrasekaran et al., 2008). This limitation motivated studies that revisited the definition of glycan conformation, extending the conformational analysis beyond the terminal sialic acid linkage to describe overall topology and dynamics of the glycan receptor upon binding to the receptor-binding site of avian and human-adapted HAs (Chandrasekaran et al., 2008; Xu et al., 2009). HA sequence determinants of preference for the “cone”-like topology of avian receptors, versus the “umbrella”-like topology of human receptors, are still being defined (Raman et al., 2014).

### C. Experimental assays to measure hemagglutinin receptor binding specificity

Experimental evidence on differential binding of avian and human viruses to sialic acid receptors in avian and human conformations, respectively, was first obtained by hemagglutination assays with erythrocytes whose surfaces had been chemically modified to display glycans terminating with either homogeneous α2,3 or homogeneous α2,6-linked sialic acids (Paulson and Rogers, 1987). Subsequent analysis of the repertoire of glycan structures in erythrocytes of various animal species informed the use of cells from different species as probes of HA receptor binding preference in hemagglutination assays (Ito et al., 1997).

Greater precision and reproducibility has been achieved with the use of purified sialylated glycans to create solid-phase binding assays with fluorogenic or enzymatic detection (Gambaryan et al., 2006; Gambaryan and Matrosovich, 1992). With these assays, it is possible to characterize the relative direct binding of whole virions or recombinant trimeric HA oligomers to glycans attached to a solid phase or the competition of such glycans with binding to a generic glycoprotein attached to the solid phase (Gambaryan and Matrosovich, 1992). In recent work, biosensor interferometry and thermophoresis have been used to measure glycan-binding avidities and affinities in a more precise manner and to relate the two (Xiong et al., 2013a). The development of glycan microarrays represented a turning point in the analysis of influenza virus receptor binding specificity, because it allowed simultaneous evaluation of virion or recombinant HA binding to a large repertoire of sialoglycans (Stevens et al., 2006; Blixt et al., 2004; Childs et al., 2009). Several measures of preference for an HA molecule or whole virus have been defined, including the ratio of the number of α2,6 to α2,3 glycans bound (Stevens et al., 2006a, 2006b) or the corresponding ratio of binding affinity or avidity (Imai et al., 2012; Xiong et al., 2013a). A limitation to predictive power is that glycans tested on current arrays may not match those present in the human respiratory tract (Walther et al., 2013). These arrays may also not present glycans in the same fashion as respiratory epithelial cells, so strategies such as measuring the binding of labelled viruses to human respiratory tissues (Chutinimitkul et al., 2010) or explant cultures (Chan et al., 2013) may be promising alternatives, although challenges remain in standardization and quantification of such assays. Structural studies of wild-type and mutant HA in complex with representative sialoglycans provide the ultimate level of detail by characterizing interactions at the atomic level. X-ray crystallography advances in recent years have accelerated structural determination, and similar progress in recombinant protein purification techniques combined with robotic crystal screening have reduced the amount of protein and labor required.

In summary, genetic and protein sequence analysis, glycan arrays, and X-ray crystallography studies provide complementary data towards understanding the sialoglycan interactions of emerging viruses, with tradeoffs of equipment and reagent costs and throughput against level of precision and detail provided.

### D. Receptor binding preference as a predictor of host adaptation of influenza viruses and pandemic risk

At present, estimating the contribution of receptor specificity to the pandemic risk posed by a novel virus relies primarily on the similarity between the receptor binding characteristics of the emerging virus and that of the most closely related HA with known transmissibility among humans or a surrogate animal model.

As noted above, hallmark residues have substantial predictive power. These distinct sets of hallmark residues in the H1, H2 and H3 subtype (Paulson and de Vries, 2013) correlate with human-adaptation in known sequences collected from birds or humans (Connor et al., 1994; Paulson and de Vries, 2013); they induce changes in receptor-binding specificity when introduced experimentally (Chen et al., 2012; Leigh et al., 1995); and experimental selection for receptor binding in vitro (Chen et al., 2012) or in ferrets (Imai et al., 2012) cause these changes to appear.

However, hallmark residue predictions of receptor-binding specificity are imperfect, as evidenced by a failure to switch in vitro receptor-binding preference from avian to human when changes observed in H5N1 strains after selection in ferret gain-of-function experiments were introduced to other H5N1 viruses (Tharakaraman et al., 2013). The involvement of other features in human adaptation, such as the topology of the bound HA-receptor complex, further complicate the genetic prediction of human adaptation, as the residues involved in these features are less well characterized (Shriver et al., 2009).

In principle, phenotypic assays that directly measure the receptor-binding preference of HA – if performed under realistic conditions that capture the interaction of the HA trimer with the receptor (Gambaryan et al., 1997; Takemoto et al., 1996; Collins and Paulson, 2004)—may better capture the trait of interest than genetic predictions of this preference. However, even here, a simple equivalence between binding preference for α2,6-linked glycans and pandemic risk could be misleading. Several viruses circulating in humans during the early phase of previous pandemics were found to show either a preference for avian receptors (Rogers and D'Souza, 1989; Connor et al., 1994) or a mixed preference for both human and avian receptors (Rogers and D'Souza, 1989; Glaser et al., 2005; Childs et al., 2009). In the case of early 2009 pandemic viruses, findings are mixed (Childs et al., 2009; Chen et al., 2011). Some of the findings of dual or avian specificity may reflect artifacts introduced when human isolates were passaged in eggs before receptor specificity was assayed; alternatively, they may genuinely reflect a transitional stage in the evolution of HA genes in human populations after transmission from other species (Connor et al., 1994; Stevens et al., 2006; Glaser et al., 2005; Stevens et al., 2010), (Table 2). Consistent with this latter possibility, an H5N1 virus isolated from a human zoonotic case in Vietnam displayed strong avian receptor preference (Yamada et al., 2006). This preference changed in the course of experiments to adapt it to respiratory droplet transmission in ferrets (Imai et al., 2012). Taken together, these findings confirm that there is a strong correlation between measured receptor preference and the host from which a virus is isolated. However, they raise questions about the predictive value of human receptor binding preference. Indeed, the examples of mixed receptor preference in human isolates from the early phase of the H1 or H2 pandemics suggest that the ability to evolve human receptor specificity over a chain of human infections, which may be present in many avian-receptor-adapted viruses, may be sufficient for pandemic emergence.

In summary, detection of a human receptor preference in a spillover virus may be an indication of increased risk, but exclusive human receptor preference is probably not necessary for an influenza A virus to initiate a pandemic. With several possible exceptions noted above, most viruses isolated to date fall within the shaded cells in Table 2, which indicates concordance between the source of the isolate and the virus trait. Thus, prioritizing pandemic countermeasures against virus lineages with inferred or measured human receptor preference will likely lead to better targeting of such countermeasures on average – that is, increase the chance of taking countermeasures against a strain that truly poses pandemic risk. However, the counterexamples of human-to-human transmission of incompletely adapted viruses (bottom left and middle cells of Table 2) suggest that in particular cases, reliance on this trait as a necessary condition to justify countermeasures may not identify all virus lineages that are in fact capable of causing a pandemic.

## Trait 2: Hemagglutinin pH of activation

### A. **Definition of the trait**

After entry into the cell, influenza viruses are internalized into endosomes, where the pH is progressively decreased. The pH of early and late endosomes, as well as lysosomes, varies between cell types, tissues, and host species. The HA must undergo a low-pH triggered conformational change to a state capable of fusing the viral and endosomal membranes. For human-adapted viruses, HA activation typically occurs between pH 5.0 to 5.5. HA variants that undergo this transition at a higher pH, as is typical for avian influenza isolates, are poorly adapted to infect human cells because the transition can happen prematurely, leading to extracellular inactivation in the mildly acidic mammalian respiratory tract (Di Lella et al., 2016; Zaraket et al., 2013a) The pH of activation can be defined as a continuous measurement representing the least acidic (highest) pH at which a particular HA molecule is triggered. Greater acid stability (lower pH of activation) is associated with greater human adaptation.

### B. **Functional, structural and genetic determinants of hemagglutinin pH of activation and its consequences**

The HA is synthesized and folded such that the fusion peptide is buried and inactive until specific activation signals are provided. The structural changes that expose the fusion peptide and lead to fusion have been described in detail (Skehel and Wiley, 2000). If the virion is exposed to sufficiently low pH outside of a host or host cell, the HA protein undergoes irreversible structural changes too early and is unable to mediate virus entry; such virions become inactivated. Thus the term *acid stability* is more broadly used to define the threshold for acidification that triggers membrane fusion (in the endosome) or inactivation (if triggered outside of the cell for an HA that is not sufficiently stable). During endocytosis, an influenza virion is exposed to sequentially lower pH values in early endosomes (pH 6.0–6.5), late endosomes (pH 5.0–5.5), and lysosomes (pH 4.6–5.0) (Mellman et al., 1986). If the HA is too stable, and fusion is not triggered in the acidic endosome of the host cell, further traffic into lysosomes results in virus inactivation by lysosomal proteases (Skehel and Wiley, 2000).

Based on surveillance studies, human-transmissible influenza isolates appear to have HA proteins that are more acid stable (have a lower activation pH) than avian influenza viruses (Russell, 2014). The HA activation pH values for H1N1, H2N2, and H3N2 seasonal viruses during the 20th Century range from pH 5.0 to 5.4 (Galloway et al., 2013). In 2009, emerging pandemic H1N1 viruses had HA activation pH values of approximately 5.5, but numerous subsequent isolates have acquired mutations that lower the activation pH to the range of the 20th Century human influenza viruses (Cotter et al., 2014; Maurer-Stroh et al., 2010; Russier et al., 2016). Broad surveys of avian and swine influenza isolates have shown that HA activation pH can vary substantially with a range from pH 4.6–6.0 (Galloway et al., 2013; Scholtissek, 1985). Among avian viruses, low-pathogenic duck viruses appear to range in acid stability from pH 5.3–6.0 and highly pathogenic avian viruses range from 5.6–6.0 (Galloway et al., 2013).

Consistent with observed patterns in natural isolates, some experimental evidence indicates that within the range of natural variation, lower activation pH is adaptive for mammalian replication while higher activation pH is adaptive for replication in avian hosts. For isolates of H5N1 highly pathogenic avian influenza virus, an increase in HA activation pH within the range of 5.2–6.0 has been associated with increased replication and pathogenicity in chickens (DuBois et al., 2011). Conversely, a mutation that decreased the HA activation pH of A/chicken/Vietnam/C58/2004 (H5N1) from 5.9 to 5.4 has been shown to attenuate virus growth and prevent transmission in mallard ducks (Reed et al., 2009) but increase virus growth in the upper respiratory tracts of mice and ferrets (Zaraket et al., 2013a, 2013b). Therefore, for H5N1 viruses, a higher HA activation pH (5.6–6.0) has been associated with a component of fitness in birds, and a lower HA activation pH (pH 5.0–5.4) has been linked to greater replication in the mammalian upper respiratory tract. Two H5N1 viruses were adapted to transmit by the airborne route between ferrets (Imai et al., 2012; Herfst et al., 2012). After a switch in receptor-binding specificity from avian to human receptors (as described above) and deletion of a glycosylation site, in both studies a final mutation that decreased the HA activation pH was shown to be necessary for airborne transmissibility in ferrets. However, these and other studies have shown that this acid stability change is not sufficient in the absence of human receptor-binding specificity (Zaraket et al., 2013a; Shelton et al., 2013). Recently, an HA protein whose activation pH was 5.5 or lower was shown to be required for the pandemic potential of 2009 pH1N1 influenza virus (Russier et al., 2016).

Nearly 100 mutations have been described to alter the HA activation pH values of various influenza A virus subtypes (Russell, 2014; Mair et al., 2014). These acid stabilizing/destabilizing residues are located throughout the HA1 and HA2 subunits and tend to be positioned in regions of the molecule that undergo large-scale changes in structure during pH-activated protein refolding (Russell, 2014; Bullough et al., 1994; Wilson et al., 1981). Mutations that modify the activation pH do not appear to alter the prefusion HA protein backbone in X-ray crystal structures (DuBois et al., 2011; Weis et al., 1990; de Vries et al., 2014). Therefore, an experimental determination or modeling of intermediate structures may be required in order to reliably predict HA pH of activation. Further complicating genetic prediction of HA activation pH values are observations that the NA and M proteins can also modulate HA acid stability in some cases (Huang et al., 1980; Su et al., 2009; Reed et al., 2010; O'Donnell et al., 2014).

### C. Experimental assays to measure hemagglutinin activation pH

A variety of experimental techniques have been developed to measure the activation pH of the HA protein, quantified as the highest pH at which the HA protein is activated to undergo the irreversible structural changes that mediate membrane fusion (Hamilton et al., 2012), or alternatively the highest pH at which, in the absence of a membrane with which to fuse, the HA protein is inactivated (inactivation pH). Classical membrane fusion assays have measured the property in bulk (Hoekstra et al., 1984). The pH of inactivation can be measured using aliquots of virions that are exposed to buffers of progressively lower pH and, after restoration to neutral pH, assayed for retention or loss of infectivity (Scholtissek, 1985). In many classical fusion assays, fluorescent probes are used to label virions, HA-expressing cells, and/or target liposomes or cells. In these in vitro assays, HA-bound target cells are typically exposed to buffers of various pH values and then lipid and/or contents mixing are measured by fluorescence (Loyter et al., 1988; Hoekstra and Klappe, 1993). Alternatively, cell monolayers expressing cleaved HA proteins can be pulsed by low-pH buffers and then incubated to readout HA-mediated cell-to-cell fusion either microscopically by syncytia formation or by reporter gene expression. If HA conformation-specific monoclonal antibodies are available for the subtype being studied, HA-expressing cells can be pulsed with low pH and then analyzed for conformational changes by flow cytometry (Reed et al., 2009). If such antibodies are lacking, HA-expressing cells can be assayed for trypsin susceptibility after low-pH exposure, with prefusion HA being resistant and postfusion HA susceptible to trypsin degradation (Steinhauer et al., 1996). Recently, methods have been developed to study HA activation and membrane fusion by individual virions, including single virion fusion using total internal reflection fluorescence microscopy (Hamilton et al., 2012).

Although the biological trigger for HA’s conformational change is a drop in pH, HA refolding can also be triggered by other destabilizing agents such as heat and urea (Scholtissek, 1985; Ruigrok et al., 1986; Carr et al., 1997). Stability at a lower pH is associated with stability at higher temperatures and higher urea concentrations, permitting the use of these agents instead of, or in addition to, pH in assays of stability. Thermal stability has been determined by measuring the threshold temperature at which denatured HA protein loses its ability to bind erythrocytes and cause hemagglutination (Linster et al., 2014).

### D. Role of hemagglutinin activation pH in pandemic risk prediction

Many questions remain regarding whether HA activation pH plays a similar role in all influenza subtypes isolated from a wide variety of avian species. For early isolates of the H1N1pdm lineage in 2009, the HA protein has an activation pH of 5.5, which appears intermediate between the canonical human (lower) and avian (higher) ranges. Subsequent H1N1pdm isolates have HA activation pH values ranging from 5.2–5.4, suggesting pH 5.5 may be the upper limit for human pandemic potential and a lower value may be preferred. Indeed, a destabilizing HA mutation in the background of H1N1pdm results in a loss-of-function of airborne transmissibility in ferrets and has been reported to be followed by re-gain-of-function by mutations that lower the HA activation pH to 5.3, a value representative of human-adapted H1N1pdm viruses (Russier et al., 2016). For the moment, it appears that while HA pH of activation that is shown experimentally to be suitable for human infection is highly typical of isolates from human pandemic and seasonal influenza (Table 3, bottom right) (Galloway et al., 2013), it is possible for humans to have symptomatic infection with (though not extensively transmit) viruses with activation pH closer to the range associated with terrestrial birds (Table 3, bottom left). Conversely (Table 3, top right), there are avian H9, H10, H14, and H15 isolates that display activation pH typical of human viruses (Galloway et al., 2013). The existence of these human-like avian viruses is perhaps unsurprising, as they may lack other essential adaptations for human transmission. As in the case of receptor binding, reliance on this trait to prioritize pandemic prevention measures should consider this property in conjunction with other properties associated with pandemic potential and will likely enrich the coverage of truly high-risk strains on average.

Systematic assessment of the predictive value of HA activation pH will require broad empirical testing, since nearly 100 residues throughout the HA molecule have been implicated in regulating HA pH of activation. Predicting activation pH from sequence will therefore require more extensive data. To address this issue, sequencing studies combined with phenotypic assays could be performed on a large range of HA variants to determine the effects of pH-altering mutations in different HA subtypes. High-resolution determination of HA structural intermediates may assist in developing molecular modeling approaches to calculate HA stability from sequence. In the interim, there is a pressing need to develop high-throughput assays for HA pH of activation, along with other properties believed important to interspecies adaptation, in the thousands of surveillance samples obtained annually.

## Trait 3: Polymerase complex efficiency

### A. Definition of the trait

The heterotrimer of influenza polymerase subunits (PA, PB1, PB2 gene products, together forming the RNA-dependent RNA polymerase) and the nucleoprotein (NP gene product) is required to transcribe and replicate the viral genome (Huang et al., 1990). The polymerase genes of viruses isolated from avian hosts show a number of genetic differences from their counterparts in viruses isolated from humans (Chen et al., 2006), and avian virus polymerase typically performs inefficiently in replicating the viral genome in human cells (Cauldwell et al., 2014; Naffakh et al., 2008). Adaptation to efficient human-to-human transmission requires efficient activity of this complex of proteins, which we refer to as the polymerase complex, in human cells (Cauldwell et al., 2014; Naffakh et al., 2008).

### B. Genetic basis of polymerase complex efficiency

**Figure 2.**
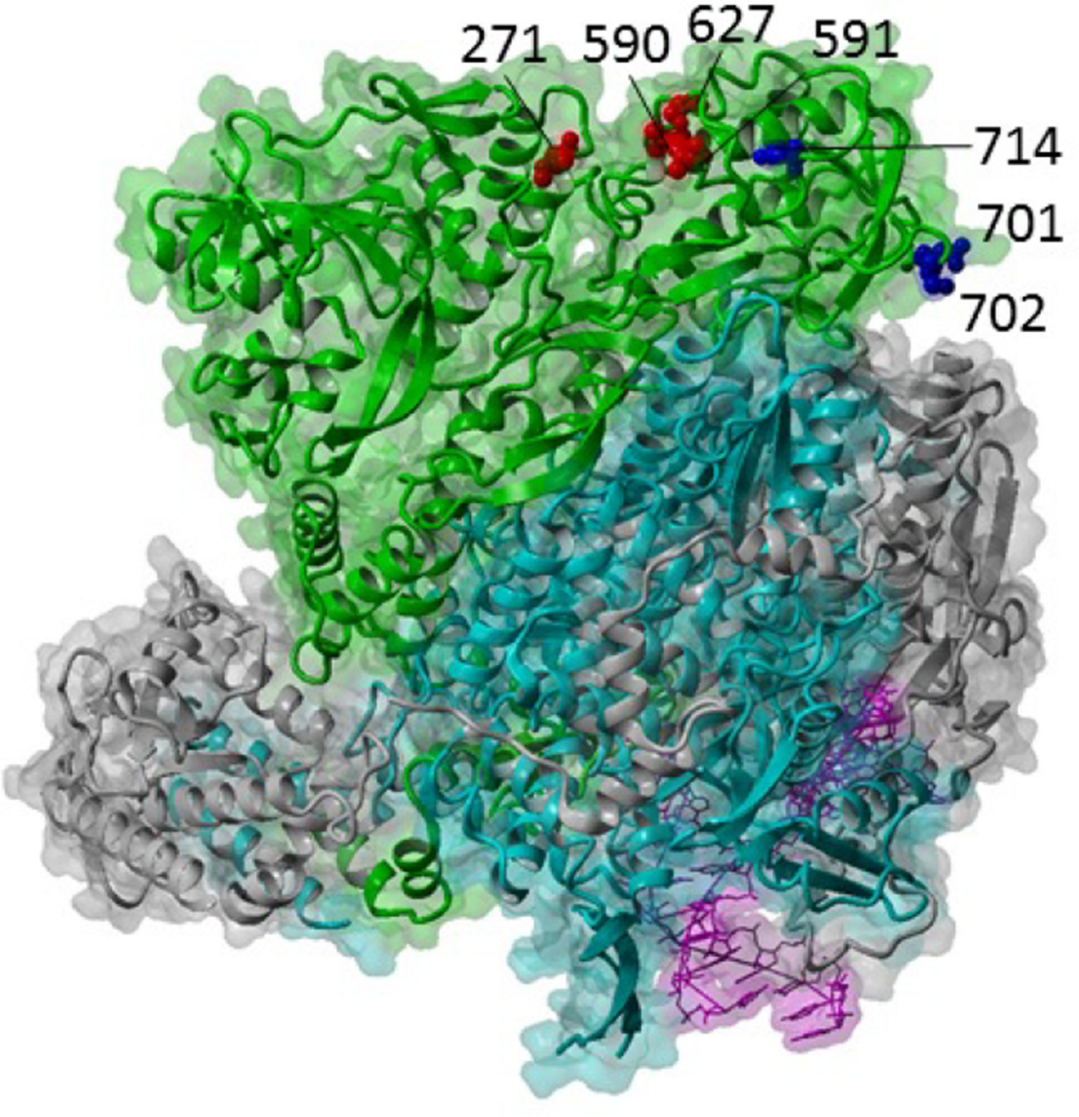
Influenza A polymerase complex from structure PDB:4WSB (Reich et al., 2014) consisting of PA (grey), PB1 (cyan), PB2 (green) and bound vRNA promoter (purple). Key host adaptation sites are indicated as red balls. Sites for importin-alpha interaction are shown as blue balls. Structure visualized with YASARA (Krieger and Vriend, 2014). **DOI:** http://dx.doi.org/10.7554/eLife.18491.010

Some mutations in PB2 are consistently associated with efficient function of the polymerase complex in mammalian cells (Figure 2). As long ago as 1977, it was shown that an avian influenza virus could achieve efficient replication in mammalian cells by acquiring mutations solely in the PB2 subunit of the viral polymerase (Spooner and Barry, 1977). The most famous of these mutations was later described as PB2 residue 627 (Subbarao et al., 1993), which is a glutamic acid (Glu) in avian influenza viruses but a lysine (Lys) in human-adapted viruses, including those that emerged in the pandemics of 1918, 1957 and 1968, and their seasonal descendants. An important exception is the virus that sparked the pandemic of 2009. In this virus, the PB2 segment had been introduced from an avian precursor into swine viruses in the 1990s, and mammalian adaptation had been achieved by a different set of PB2 mutations including changes at residues at 271, 590 and 591 (Mehle and Doudna, 2009). Now that the 3-dimensional structure of the viral polymerase has been elucidated, we can see that residue 627, 271, 590 and 591 lie on the same external surface. Mammalian-adapting mutations increase the positive charge of this domain, suggesting that they either adapt the virus for interaction with an enhancing host factor or enhance its ability to repel a restriction factor (Mehle and Doudna, 2009). Recently a host factor, ANP32A, that differs between mammals and flighted birds was shown to be a cofactor of the influenza polymerase, and the species specific difference could explain the inefficient function of avian virus polymerase and the stringent selection for the 627Glu->Lys adaptive mutation in mammals (Long et al., 2016).

Another residue implicated in mammalian adaptation of the polymerase is residue 701 of PB2, which lies close to but is distinct from the 627 cluster. It has been suggested that this mutation and others in this domain at residues 702 and 714 affect the interaction between PB2 and importin-alpha isoforms either in a way that enhances nuclear import of newly synthesized PB2 or that affects polymerase function once inside the nucleus, the site of viral RNA replication (Resa-Infante et al., 2008; Gabriel et al., 2005; Sediri et al., 2015). Other mutations have been described that adapt PB2 for the mammalian nucleus (for example the triplet threonines at positions 147, 339 and 588) but whether they affect interaction with ANP32A, importins or as yet unidentified host factors is not yet elucidated.

The adaptive value of these mutations is shown by experimental or observational data in which a mammalian host is infected with a virus whose PB2 is not adapted for efficient mammalian replication, but such a mutation becomes common in the virus population over the course of infection. Such evolution has been observed in a fatal human case of influenza A/H7N7 (Jonges et al., 2014) and in mouse experiments following serial lung passage using an isolate from this outbreak (de Jong et al., 2013). Lys at position 627 has also been associated with greater severity in zoonotic H7N9 (Sha et al., 2016) and H5N1 (de Jong et al., 2006) cases However, reverse genetics experiments show that certain strains of avian influenza may be less able to accept these mutations than others (Long et al., 2013).

### C. Experimental assays to measure polymerase complex efficiency in human cells

Polymerase complex efficiency in human cells can be measured by an in situ assay in which the influenza polymerase is reconstituted from cloned cDNAs in plasmids and then coexpressed with “minigenome,” a viral-like RNA encoding a reporter, such as luciferase. By measuring the rate of reporter accumulation in the transfected human cell line, specific combinations of RNA sequences for the polymerase-complex viral genes can thereby be screened directly for their efficiency in producing the mRNA encoding the reporter gene product, providing a measure of human adaptation of the polymerase complex (Moncorgé et al., 2010).

The original form of the in situ reconstituted polymerase assay requires expression of just the minimal set of four viral proteins to replicate the minigenome RNA: PB1, PB2, PA and NP. However, recent work showed an important additional role for another protein, the nuclear export protein (NEP), which is translated from a spliced mRNA derived from RNA segment 8 (that also encodes the major interferon antagonist NS2) (Robb et al., 2009). In human H5N1 isolates that do not contain PB2 host-adapting mutations, the inefficient activity of these avian polymerases in human cells could also be compensated for by certain mutations in NEP (Mänz et al., 2012). It appears that NEP is an important regulator of the balance between transcription and replication ([Bibr bib33][Bibr bib150]), and can thus enhance fitness in viruses containing otherwise inefficient polymerases. The mechanism of this is as follows: the polymerase-enhancing domain of NEP is masked when NEP is folded in one conformation. However, mutations that increase the ability of NEP to rescue avian polymerase function allow more ready unfolding of the protein, unmasking the “activating” domain at the lower temperature of the mammalian respiratory tract. Interestingly, NEP overexpression in cells in which human-adapted polymerase is reconstituted is inhibitory because excess complementary RNA accumulates at the expense of messenger RNA and further viral RNA replication (Robb and Fodor, 2012). Thus although a short-term adaptation of avian virus polymerase to mammalian cells can be achieved in this way, it may be that further compensatory changes rebalance NEP function in the face of polymerase adaptation during continued circulation in humans, although direct evidence for this selection is lacking. Indeed, although the rescue of low polymerase activity by NEP may explain the human infections by H5N1 viruses that lack other polymerase adaptations, it is not clear that such rescue is sufficient to create a level of transmissibility consistent with pandemic spread. Nonetheless, this finding shows that the minimal polymerase assay is not always sufficient to predict viruses that have functionally adapted polymerase activity to human cells and that a role for other viral proteins including at least NEP should also be considered in assessment of polymerase function.

Alternatively, polymerase activity could be measured in the context of viral infections (although this will require proper containment). This could be achieved by measuring intracellular levels of viral transcripts using transcriptomics or qRT-PCR. Such experiments would provide important information if they are performed using appropriate cell lines (or respiratory explants) at the temperature of the human airway (33°C). It has been suggested that plaque size at 33°C can be used as a surrogate measure of polymerase function but plaque size is a mutligenic trait. The predictive value of such assays for transmissibility is limited.

Ultimately, it would be valuable to develop a simple screen to assess the ability of a viral polymerase to support replication and transmission in humans. This phenotype is influenced by at least 4 different viral genes and involves interactions with several different human host factors. If all the relevant host factors were enumerated, one could imagine quickly converting sequence information into an assay that tested for interactions that should support activity. Along these lines the recent description of a host factor, ANP32A that differs between flighted birds and mammals and explains the poor activity of avian polymerase in mammalian cells is a step forward (Long et al., 2016).

### D. Role of polymerase complex efficiency in pandemic risk prediction

The inefficient polymerase of avian influenza viruses in mammalian cells is one of the host-range barriers that likely diminishes pandemic risk. Unlike the requirement for adaptive mutation in the novel HA, this polymerase barrier can be rather readily overcome by reassortment in which an avian virus with novel antigenicity can acquire one or more polymerase genes from mammalian-adapted viruses. In addition, adaptation of avian virus polymerase by accumulation of adaptive mutations in either the polymerase genes or possibly in other viral genes such as the NS segment encoding NEP can enhance avian virus polymerase function sufficiently to support a host range jump.

Many H5N1 viruses that circulate today in the avian reservoir already have mutations in PB2 at 627 (Long et al., 2013) or 701 (de Jong et al., 2006), likely resulting from the reintroduction of mammalian-adapted strains back into the wild bird reservoir. These have been associated in human infections with more severe cases (de Jong et al., 2006). The fact that these strains have not achieved sustained human-to-human transmission demonstrates that while polymerase adaptations to humans are likely necessary, they are not sufficient for a strain to spark a pandemic. Moreover, the absence of the signature PB2 627K mutation in the 2009 H1N1 pandemic strain demonstrates the limitations of relying on any single mutation for risk prediction (Herfst et al., 2010); viruses with the avian-like residue have also been isolated from zoonotic human cases of H5N1, H7N9, and H9N2 infections (Table 4, bottom left). On the other hand, the concept that adaptation of the polymerase is necessary for sustained human transmission is validated by findings that the 2009 pandemic strain had adapted to replication in human cells by changes other than E627K within the polymerase (Mehle and Doudna, 2009). Identification of biophysical mechanisms common to mammalian-adaptive mutations may in the future provide the basis for new biological or biophysical assays of polymerase function to inform risk predictions.

In summary, no single polymerase mutation appears to be predictive of pandemic risk for all viruses, but the concept that the polymerase must adapt to human cells before it can cause extensive human-to-human transmission appears consistent with the four pandemic jumps that have occurred in modern times.

## Discussion

There has been tremendous progress in understanding the traits involved in the adaptation of avian influenza viruses for efficient human-to-human transmission and the genetic and structural basis of each of these traits. While the ability to use virus sequence data to inform risk assessment of pandemic potential is improving, it remains essential to consider these data alongside other experimental and epidemiological data. For example, in 2013 there was a substantial increase in the number of human infections with A/H5N1 viruses in Cambodia. The increase in infections was cause for substantial concern by itself. Enhancing the level of concern was the finding that some of the viruses collected from infected humans contained previously identified genetic mutations suggestive of human adaptation (Davis et al., 2014). These findings led to extensive epidemiological and experimental investigations and then to the decision to produce a candidate vaccine virus from a virus representative of the 2013 Cambodian outbreaks.

While predictions of virus phenotypes from sequence data can be informative, they are not infallible, for several reasons, notably the large number of sites involved in determining such traits (Raman et al., 2014; Russell, 2014; Cauldwell et al., 2014; Mehle and Doudna, 2009; Mänz et al., 2012; Herfst et al., 2010), the important role of epistasis (dependence of a mutation’s effect on the genetic background in which it appears) in determining these traits (Russell et al., 2014; Kryazhimskiy et al., 2011; Gong and Bloom, 2014; Bloom et al., 2010; Wu et al., 2016; Raman et al., 2014; Tharakaraman et al., 2013; Gong et al., 2013), and the consequent imperfections in our ability to map single sequence polymorphisms to a trait value. For example, the hallmark HA amino acid residues 190, 226 and 228 are important to human adaptation, but “human-adapting” mutations at these residues do not always change receptor-binding specificity; it depends on the genetic background. Similarly, amino acid residues 627 and 701 of the PB2 protein are often involved in human adaptation, but both carried the “avian-adapted” residue in the 2009 H1N1 pandemic strain. When these changes were introduced individually or together in the laboratory, the resulting polymerase showed greater activity in a minigenome assay, but replication was unchanged or attenuated in vitro, in mice, and in ferrets (Herfst et al., 2010; Jagger et al., 2010). After the pandemic strain was identified and its anomalous residues at these sites noted, other sites within PB2 were identified and found to be responsible for human adaptation (Mehle and Doudna, 2009; Yamada et al., 2010).

Based on the evidence to date, it seems clear that all three of the traits considered in this review, and possibly others in Table 1, must be simultaneously present at least to some degree for a strain to cause a pandemic. Yet with only a few instances of pandemic strains emerging per century, it should not be surprising that a new pandemic strain would violate an apparent rule of human adaptation that applied perfectly to previous pandemic strains, as in the case of the PB2 residues associated with human adaptation in 2009, or as in the case of activation pH of HA in early 2009 isolates (Russier et al., 2016), which had a value outside the range previously seen in human influenza viruses. It is unclear whether the list of sites and phenotypic traits associated with human adaptation is nearly complete or will continue to grow as we experience additional pandemics. At least for the traits of receptor binding (Childs et al., 2009) (Table 2) and acid stability (Russier et al., 2016) (Table 3), full human adaptation may not be required to initiate a pandemic in a virus that is otherwise well-adapted for humans. Thus, whether or not the list of traits required for pandemic is now complete, our understanding of where the threshold lies for being sufficiently human-adapted continues to change.

There are three complementary approaches to address these limitations: improving genetic prediction of biological traits, improving assays of these traits, and improving animal models of human transmission; all approaches are currently progressing in parallel (Holder et al., 2011; Simon et al., 2011). The first approach aims to further refine our understanding of sequence-to-trait relationships by continued studies of diverse, naturally or artificially produced mutations and their effects on the traits of interest. Such research could use all of the approaches described above and higher-throughput assays that could be developed with improved technology, for example as described in Box 3. This will include generating mutations not found in known strains in nature to probe for those that could be involved in human adaptation in the future (Hanson et al., 2015; Thyagarajan and Bloom, 2014). The goal would be to identify classes of functionally equivalent substitutions, sufficient individually or in defined combinations to confer a trait of interest when introduced into a defined, avian-adapted genetic background. Use of in vitro approaches with noninfectious viruses or viral components, or infectious viruses containing surface proteins to which there was already population-wide immunity would reduce the possible biosafety and biosecurity risks of such studies (Lipsitch and Inglesby, 2014).

The second approach is to develop and improve the throughput and accuracy of biochemical and cell-biological assays of these traits, so that virus isolates can be characterized phenotypically in a routine manner, reducing reliance on sequence-based predictions. It seems feasible to develop high-throughput versions of many of the assays described in this review for each of the three traits discussed, which could then be routinely run on surveillance isolates to contribute to risk prediction. For none of these three traits is there a single gold standard assay, and different assays may provide different estimates of risk.

The third approach is to improve animal models to more precisely study phenotypes that are important for human adaptation, and to clarify whether the notion of 'mammalian adaptation' is in fact a valid category. Ferrets are the closest known model for human transmission (see Box 4). Respiratory-droplet transmission studies in ferrets, and potentially in other animal models, have shown a remarkably strong correlation with human transmissibility of influenza A strains (Buhnerkempe et al., 2015). While these assays are not perfectly predictive, they may be the most reliable way at present to assess the transmission potential of a virus in human populations. Here a partial counterexample to their overall strong predictive value is H7N9 avian influenza isolates from human zoonotic cases. These viruses transmit in ferrets, albeit less efficiently than human seasonal strains, yet human-to-human transmission has been extremely rare in the hundreds of human zoonotic cases caused by H7N9 (Buhnerkempe et al., 2015). A challenge is the expense and practical challenge of using large enough numbers of ferrets (Buhnerkempe et al., 2015; Belser et al., 2013; Nishiura et al., 2013) to assess transmissibility; this will remain a technique of limited throughput for the foreseeable future. Nonetheless, the value of ferret testing for risk assessment can be enhanced in at least two ways: first, by standardizing the conditions for ferret transmission experiments, so these can be more confidently compared between laboratories; and second, by continuing to combine ferret studies with studies of viral traits and sequence/structural studies to further identify correlates of transmissibility in ferrets.

10.7554/eLife.18491.011

Box 4. **Ferret model: validity and limitations in pandemic risk assessment.**

The use of small mammalian models in influenza virus pathogenesis and transmission has proven invaluable for the study of these complex, polygenic traits. The ferret model is particularly valuable, as ferrets are highly susceptible to most influenza A viruses without the need for prior host adaptation. However, even this gold-standard model is not a true substitute for humans. Below, we summarize the benefits, drawbacks, and alternatives to the ferret model for the study of influenza.

*Validity*. Influenza is a respiratory pathogen in humans, and employing mammalian models that possess comparable lung physiology permits a greater extrapolation of results from the laboratory. Importantly, the linkage types and distribution of sialic acids throughout the ferret respiratory tract are generally comparable to humans (Jayaraman et al., 2012; Jia et al., 2014): like humans, ferrets express the sialic acid ​N-acetylneuraminic acid (​Neu5Ac), but not the sialic acid ​N-glycolylneuraminic acid (​Neu5Gc), on respiratory epithelia. As a result, ferrets are uniquely suited for the study of influenza viruses compared with other small mammalian models which express Neu5Gc (Ng et al., 2014). Furthermore, human and avian influenza viruses exhibit comparable binding to upper and lower respiratory tract tissues in ferrets and humans (van Riel et al., 2006; Shinya et al., 2006).

Secondly, ferrets infected with influenza viruses demonstrate numerous clinical signs and symptoms of infection associated with human disease. Ferrets infected with human influenza viruses often exhibit transient weight loss, transient fever, and sneezing, whereas infection with selected HPAI viruses in this species can lead to pronounced weight loss, sustained fever, lethargy, dyspnea, and neurological complications (Belser et al., 2009). Thus, ferrets represent a preclinical model to assess the ability of novel vaccine and antiviral treatments to mitigate influenza virus. As ferrets are a suitable model for the coincident study of pathogenesis and transmission, this model allows for a greater understanding of virus-host interactions and the interplay between both of these parameters.

Finally, the ferret model can yield valuable insights about the potential human-to-human transmissibility of influenza viruses – the critical determinant of pandemic risk. A recent meta-analysis showed that estimates of transmissibility derived from ferret respiratory droplet transmission studies could explain 66% of measured variation in human transmissibility, for influenza subtypes that have been detected in humans (Buhnerkempe et al., 2015). Furthermore, there is a strong statistical relationship between the attack rates measured in particular ferret experiments and the probability that the influenza strain in question is capable of sustained transmission among humans: if two-thirds or more of contact ferrets become infected via respiratory droplets, then the strain is likely to have pandemic potential (see figure). However, extrapolation of this relationship to novel strains is inherently risky, and variable outcomes observed for H7N9 influenza transmission in ferrets highlight the potential for false alarms. Further analysis of ferret transmission experiments, ideally in concert with molecular and virological research, could raise their sensitivity and specificity for identifying pandemic threats.

*Limitations.* There is no ‘perfect’ small mammalian model for influenza. A longstanding challenge of the ferret model has been limited availability of ferret-specific commercial reagents compared with other models, though recent sequencing of the ferret genome should improve this situation (Peng et al., 2014). Ethical considerations, and the size and cost of ferrets, necessitate generally small sample sizes in ferret experiments, limiting statistical power (Belser et al., 2013). Like other vertebrate models, the ferret is not appropriate for high-throughput screens, so research in the ferret model is most potent when complemented with in vitro and computational approaches. Finally, ferrets are not well suited to model the multiple influenza exposures over several years that may be experienced by humans and may mold their immune responses in ways that affect the infection risk with subsequent viruses (Andrews et al., 2015). Studies of first influenza infection in ferrets may thus overestimate infection and/or transmission risk relative to that in populations with a history of prior infection with related viruses (Belser et al., 2016).

*Alternatives.* The ferret is but one of several well-characterized mammalian models for influenza virus. Mice are widely used in the field as they offer a greater availability of commercially available species-specific reagents, permit studies with greater statistical power due to larger sample sizes, and offer the advantage of transgenic animals. However, not all human influenza viruses replicate well in mice without prior adaptation due to a predominance of avian-like receptors in the murine respiratory tract; also mice do not display clinical signs and symptoms of influenza that mimic humans, and are not a reliable model for virus transmission studies. The guinea pig is another model, and offers several comparable advantages to ferrets, including generally similar lung physiology to humans and potential for transmission studies. Experiments in guinea pigs are often less expensive than in ferrets, because of lower husbandry costs and reduced drug costs when dosing is based on body weight (Lowen and Palese, 2007). However, guinea pigs do not exhibit clinical signs and symptoms of infection similar to humans, and do not exhibit severe disease following infection with HPAI or pandemic influenza viruses, limiting their utility for viral pathogenesis studies.

**Box 4-figure 1.**
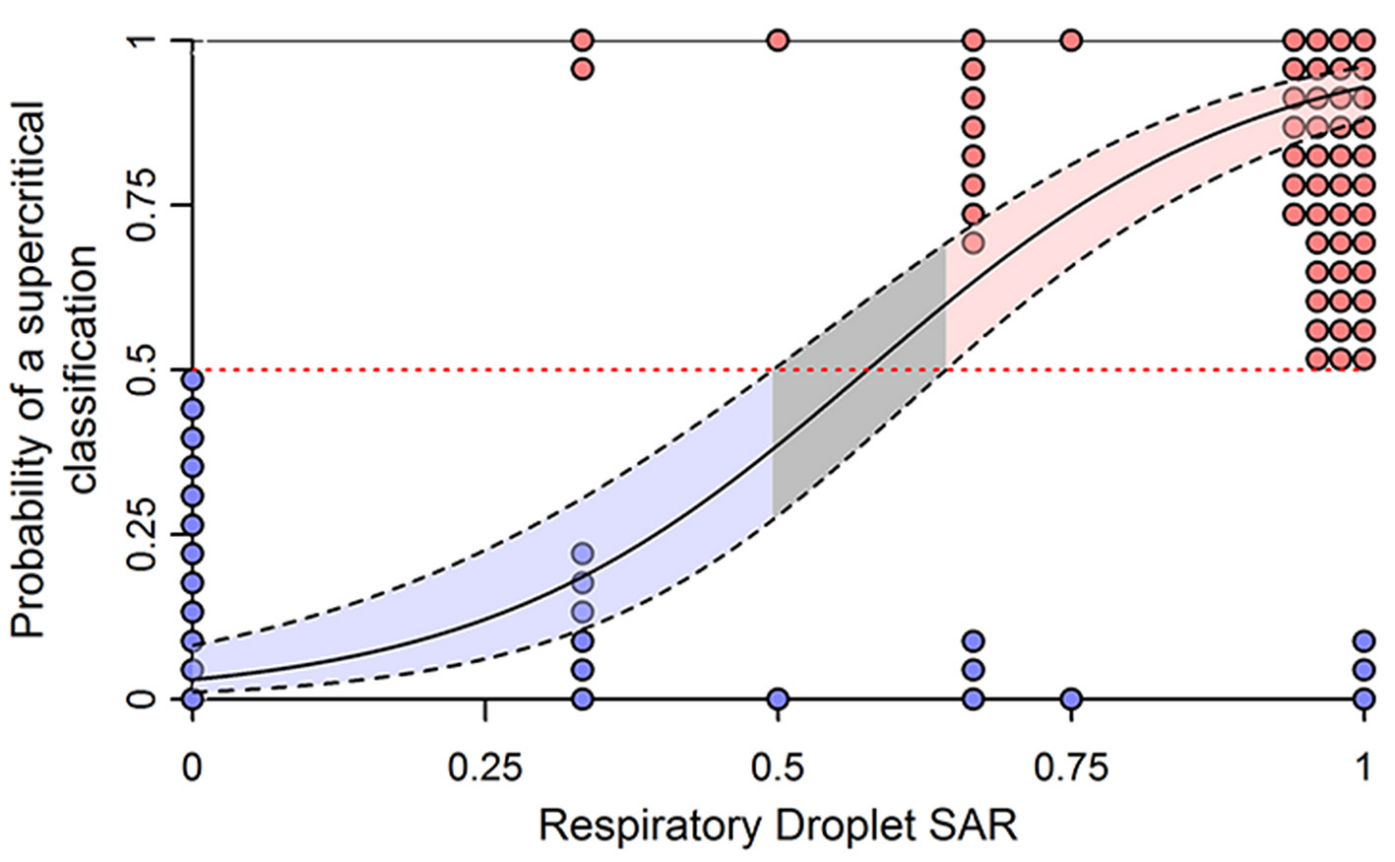
Ferret respiratory droplet transmission experiments predict the potential for sustained human-to-human transmission of influenza viruses. The solid line shows the weighted logistic regression relationship predicting the probability that a given strain is supercritical (i.e. capable of sustained spread among humans), and the dashed lines show the 95% confidence interval for the prediction. Filled circles show the measured secondary attack rates (SAR) in ferrets for influenza subtypes that are known to be subcritical (blue) or supercritical (red) in humans. The filled pink area shows the range of SAR for which the virus is significantly likely to be supercritical. Reprinted from (Buhnerkempe et al., 2015). **DOI:** http://dx.doi.org/10.7554/eLife.18491.012

**DOI:** http://dx.doi.org/10.7554/eLife.18491.011

While the biological properties of a virus certainly play a large role in determining the pandemic risk posed by a strain, it is possible that even a virus perfectly adapted for human-to-human transmission might fail to transmit extensively, due to ecological factors, chance, or both. Initiation of a pandemic requires not only a well-adapted virus but ecological opportunity to spill over into humans (perhaps multiple times if the first introduction is not “successful” [Mills et al., 2006]), as well as a human population that is immunologically susceptible and sufficiently connected to establish ongoing transmission (Box 5). Additional complexity arises from the fact that selection pressures for within-host proliferation and competition may diverge from those needed for efficient transmission (Park et al., 2013). Genetic bottlenecks at the time of transmission (Poon et al., 2016; Varble et al., 2014; Zaraket et al., 2015) may further enhance the role of chance, as a highly adaptive variant arising in a host may not get transmitted in a particular event. However, selective bottlenecks, which have been observed in experimental transmission of H5N1 and avian-like H1N1 viruses in ferrets, could lower the barrier to emergence of human-adapted viruses (Moncla et al., 2016; Wilker et al., 2013). Both ecological and host factors are considered in the CDC’s IRAT (Trock et al., 2015).

10.7554/eLife.18491.013

Box 5. **Role of seroepidemiology in pandemic risk assessment.**

Pandemic threat assessment can also be enhanced by immunological surveys of human populations in geographical area where strains of concern are known to be circulating (Van Kerkhove et al., 2011). Serological surveys can help to estimate the frequency of spillover infections from non-human to human hosts and also to assess the degree of cross reactivity arising from endemic human strains that share recent genomic ancestors with non-human strains of concern (Figure for Box 4).

Attempts have been made to use serological surveys to estimate the rate of spillover infections to humans for recent strains of concern (Wang et al., 2012; Van Kerkhove et al., 2012). Sometimes blood samples are obtained from the general population (Chao et al., 2011) and other times only high risk individuals are tested. Inherent measurement error and cross-reactivity between human and non-human strains make the measurement of low rates of incidence problematic (Van Kerkhove et al., 2012). Confidence that serologic responses truly reflect zoonotic transmission, rather than cross-reactivity with antibodies generated in response to human influenza infection, may be enhanced by comparison of high-risk persons to those without known exposure to zoonotic sources (Huang et al., 2015; Gomaa et al., 2015). Although there is evidence of exposure of poultry workers to H5N1 influenza viruses in China, rates are much lower than for other endemic non-human influenza viruses(Kim et al., 2011), such as H9N2 (Blair et al., 2013). More recent studies of exposure of high risk workers to the H7N9 lineage suggest even higher rates of exposure to this new strain than has been observed in similar studies of H5N1 or H9N2 (Wang et al., 2014).

Even when rates of spillover can be estimated accurately, the use of such information in pandemic threat assessment is not obvious. Clearly, the first detected presence of human infections for a given strain is of concern because the degree of transmissibility among humans is unknown. Should the emergent strain fail to achieve sustained transmission, it is not immediately clear how best to use further information on the frequency of human spillover infections. For example, should we interpret high sustained levels of human spillover as evidence of increased risk because of the number of human infections, or as evidence of decreasing risk because of the number of times the strain has failed to achieve sustained transmission?

Cross reactivity between non-human and human influenza strains has implications beyond the measurement of spillover infections. Often levels of cross reactivity in humans may indicate some degree of reduced population susceptibility (Worobey et al., 2014). All else equal, such evidence of lower population susceptibility should reduce our level of concern about a pandemic threat from a particular virus, because even if it gains efficient human-to-human transmissibility, its effective reproductive number and the proportion of the population at risk will be less than for a virus to which there is no cross-reaction in the population. For example, older individuals are thought to have been far less susceptible to pandemic H1N1 than were younger individuals, because they had previously been exposed to similar strains early in life (Yu et al., 2008). The low average age of infection with a swine variant form of H3N2 (H3N2v) in North America (Jhung et al., 2013) is likely driven by reduced susceptibility in adults because of early exposure to similar strains. Such immunological overlaps are likely to be a general feature of influenza emergence because human strains frequently emerge into swine populations (Nelson et al., 2015).

Data on reduced human susceptibility due to cross-reactivity must be synthesized with other data used for threat assessment. In some cases, the aging of the part of the population with prior exposure to a closely related strain could be the most important known factor increasing the risk of an emergence event. Mechanistic models could be used to estimate the degree of increased risk of emergence due to the aging of partially immune cohorts.

**Box 5-figure 1.**
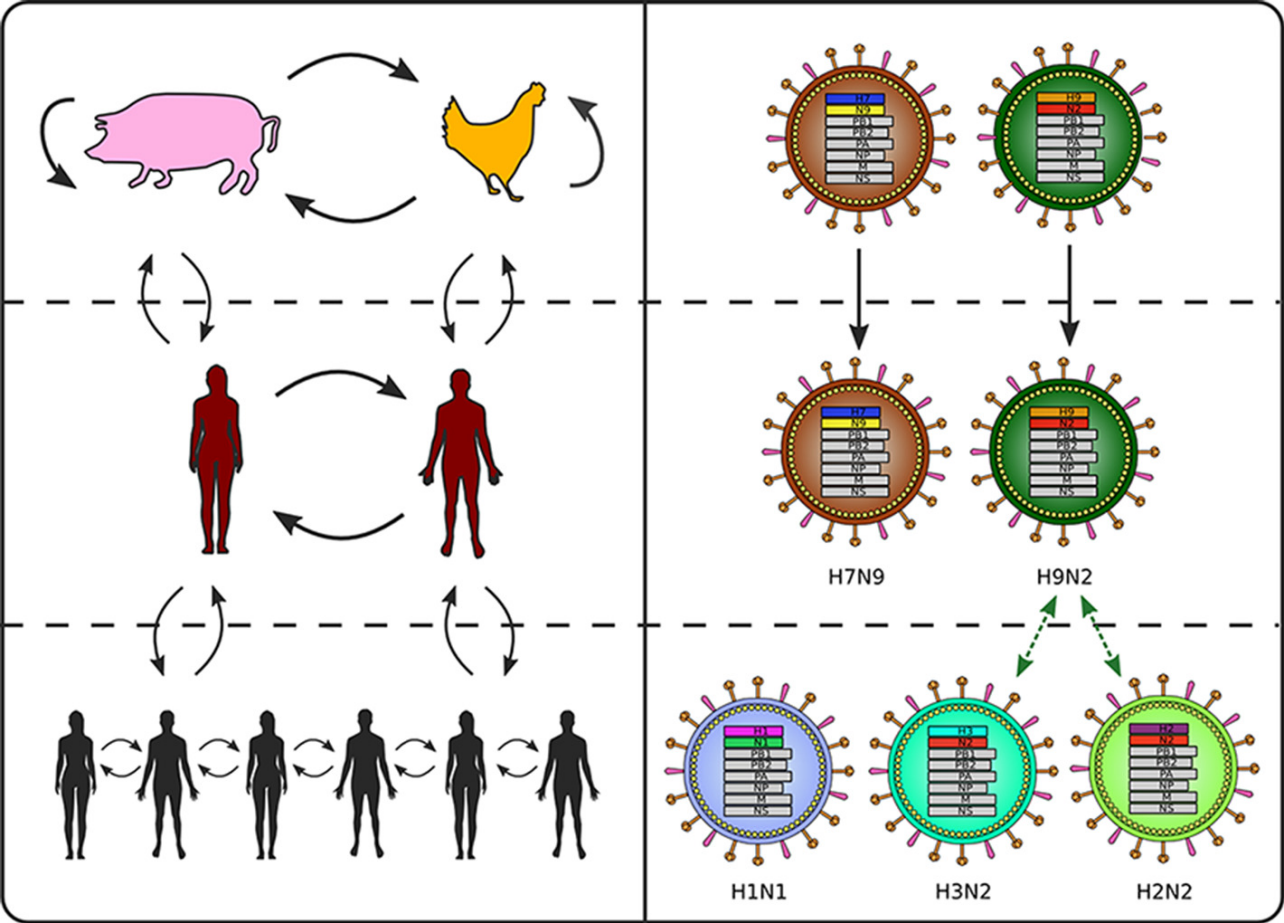
Transmission genomics of non-human transmission (top), spillover transmission (middle) and sustained human transmission (bottom). Haemagglutinin and neuraminidase gene segments have been color-coded to show an example shared infection history in humans who are current spillover hosts for H7N9 and H9N2. These shared evolutionary histories make it challenging to interpret serological studies of human spillover infections. Humans infected by H2N2 or H3N2 will likely have cross-reactive antibodies to H9N2, because of the similarity between the neuraminidase in those viruses. Because incidence of spill-over infection is likely to be low, even low-levels of cross-reactivity can make the interpretation of serological studies of the general population challenging. **DOI:** http://dx.doi.org/10.7554/eLife.18491.014

**DOI:** http://dx.doi.org/10.7554/eLife.18491.013

Evolutionary factors also play a role in pandemic risk evaluations. Even with excellent surveillance, we may never isolate exactly the virus that is destined to cause a pandemic from an animal reservoir or a zoonotic human case; rather, we may isolate its evolutionary precursor. Understanding the potential of a strain to produce pandemic-capable progeny is yet a further scientific challenge. Perhaps the most startling finding of the gain-of-transmissibility experiments with H5N1 avian influenza viruses was that so few mutations were required to convert a strain circulating in birds to mammalian transmission. This concern was reinforced by a finding that many of these mutations, including combinations of some of them, were already present in strains isolated during surveillance (Russell et al., 2012). The interpretation of the latter finding, however, is complicated by the problem of epistasis: the effect of these mutations in the genetic background of field strains may or may not be the same as in the strain studied in the laboratory.

It seems clear that a pandemic risk assessment informed by genetic sequence data is better than one uninformed by such sequence data, but the thought experiment of considering the 2009 swine-origin virus, had it been seen prior to initiating the pandemic, shows that such efforts may fail to identify the risk posed by strains that subsequently cause a pandemic. According to the knowledge at the time, early human isolates of 2009 (and presumably their swine precursors) would have had an HA intermediate between human and avian adaptation in terms of receptor binding. They had an activation pH outside the range previously seen in human viruses and more typical of avian viruses. Moreover, these viruses lacked the amino acid residues then thought to confer human adaptation for the polymerase complex. We must imagine that had this strain been detected in swine surveillance prior to the pandemic emergence, genetic as well as phenotypic considerations would have marked it as low risk, creating a false-negative risk assessment. Given that this virus did in fact create a pandemic, it is evident that failure of a nonhuman influenza virus to fully meet the three criteria discussed in this review does not disqualify it from posing a significant risk of a pandemic.

Whether false positive predictions of high pandemic risk are also possible is more difficult to determine, because even a strain that is truly high risk may fail to cause a pandemic for any number of reasons; thus it is challenging to prove that an assessment of high risk for a particular strain was erroneous. From a decision-making perspective, a false positive is perhaps less worrisome than a false negative, as a false positive may motivate expenditure on prevention measures directed at a strain that would not have caused a pandemic, while a false negative may lead to a failure to respond to a strain that would.

As we seek to improve our understanding of genetic and phenotypic bases of efficient human transmission of influenza viruses, there are multiple possible approaches. One approach that has received considerable attention recently is to perform gain-of-transmissibility studies in highly pathogenic avian viruses; this has been controversial because of concerns about the unusual biosafety and biosecurity risks entailed in such studies (for contrasting perspectives on these risks, see the exchange in 2014–5 between Lipsitch and Inglesby, and Fouchier [Lipsitch and Inglesby, 2014, 2015; Fouchier, 2015]).

There are alternative approaches to ferret gain-of-transmission experiments in highly pathogenic avian influenza viruses, though disagreement remains about the level of evidence such alternatives provide. One alternative is to perform similar experiments starting from avian viruses that are not highly pathogenic in mammals, and/or are related to currently circulating human seasonal viruses, so that immunity would already be present in the population. Such experiments can provide the same degree of causal rigor as gain-of-function in highly pathogenic avian viruses with novel surface antigens and can elucidate general principles of mammalian adaptation, but they cannot confirm that the same changes would be observed in other strains that are not used in the experiment. A recent report shows a related way forward: the recreation of the steps of mammalian adaptation using viruses whose HA and NA are already circulating in humans (Lakdawala et al., 2015). Such loss+gain-of-transmissibility experiments reconstruct the properties of naturally occurring seasonal human strains, from laboratory-generated, avian-adapted (or at least human-deadapted) precursors. Reconstructing such seasonal strains should pose a risk similar to that of working with the seasonal strains themselves, less than that of a novel subtype. A 2011 report employed a similar strategy, demonstrating the importance of HA activation pH in mouse adaptation by selection experiments on a live attenuated H5N1 vaccine strain lacking the NS1 gene (Krenn et al., 2011). More recently, a 2009 H1N1 pandemic virus was modified to express a mutation that increased pH of HA activation, then selected in ferrets for droplet transmission, and it was found that a second site mutation partially restored the lower pH of activation of the selected virus (Russier et al., 2016). One limitation of de-adaptation strategies is that the acquisition of transmissibility is perhaps most likely to evolve by reversion of the de-adaptation changes. As with all gain-of-function and loss-of-function studies, epistatic effects of other loci in the genetic background of the viruses used in such studies set limits to the generalizability of such experiments. Another kind of alternative is simple characterization of ferret transmissibility of naturally occurring highly pathogenic strains without selection for airborne transmission. This approach can provide correlative evidence for the importance of genetic differences but cannot prove the mechanistic role of any particular change.

Even if strain-based assessment methods were much better, surveillance would be a key rate-limiting step for pandemic risk assessment to direct countermeasure development. If a virus about to cause a pandemic is not found in surveillance it cannot be assessed. The fact that we have yet to identify a pandemic strain in nonhuman hosts or in human spillover cases before the pandemic starts indicates there is much work to be done. Although surveillance has expanded since the 2009 pandemic, it has not been designed to optimize the chances of detecting a pandemic strain before it becomes pandemic; indeed, how to do so is not clear at present. Some possible considerations would be to maximize the diversity of isolates collected, to preferentially sample strains that are known to cause human infections, and to feed back information from risk assessments to inform choice of sampling. Rapid sequencing and phenotypic characterization of strains and dissemination of this information, along with interpretations of the risk profiles implied, is also important to maximizing the value of surveillance. Further thought should be given to the possibility of using high-throughput sequencing as a screen for which viruses should be subjected to phenotypic testing, which for the moment is typically more costly, slower, and lower-throughput than sequencing. More deliberate approaches to the design of surveillance systems would also depend on answering the question addressed in Box 2: how different must a virus be from previously characterized viruses to merit separate evaluation of its pandemic risk? The uncertainties noted above about the phenotypic characteristics of viruses isolated from previous pandemics (which may have been present in the primary isolate or may have arisen during egg passage in the laboratory) underline the need for careful attention to passage histories of surveillance isolates to avoid altering their genotype and phenotype post-isolation (Bush et al., 2000). Expanding and rationalizing surveillance in this way would require overcoming political, logistical and financial constraints that vary between countries and regions.

Even with all of the foregoing suggestions in place, it may be improbable that we can reliably identify the 'needle in the haystack' that is the next pandemic influenza strain. Ultimately, the goal is not risk assessment for its own sake, but preparedness and early response to pandemic threats. In other areas where security is at stake, it has been argued that making and improving predictions should be accompanied by a systematic effort to design responses that will not fail even if the predictions are wrong (Danzig, 2011). In the influenza context, the value of some countermeasures is strongly reliant on our ability to identify truly high-risk prepandemic threats: notably, preparation of seed vaccine stocks for candidate pandemic strains, stockpiling of subtype-specific vaccines, and culling of poultry infected with such strains. Other types of countermeasures, ranging from strengthening local public health departments to stockpiling antivirals or ventilators to developing faster processes for vaccine manufacture to universal vaccines that should be effective against any influenza A strain, should provide benefits whether or not we have advance notice of the strain causing the next pandemic. A comprehensive assessment of priorities to prevent or mitigate the next influenza pandemic should consider the balance between improving our risk assessment capacity and developing responses robust to the possibility that we will once again be caught by surprise.
